# Protocellular CRISPR/Cas‐Based Diffusive Communication Using Transcriptional RNA Signaling

**DOI:** 10.1002/anie.202202436

**Published:** 2022-04-26

**Authors:** Shuo Yang, Alex Joesaar, Bas W. A. Bögels, Stephen Mann, Tom F. A. de Greef

**Affiliations:** ^1^ Institute for Complex Molecular Systems Eindhoven University of Technology P.O. Box 513 5600 MB Eindhoven The Netherlands; ^2^ Centre for Protolife Research and Centre for Organized Matter Chemistry School of Chemistry and Max Planck-Bristol Centre for Minimal Biology School of Chemistry, University of Bristol Bristol BS8 1TS UK; ^3^ School of Materials Science and Engineering Institute of Molecular Medicine (IMM) Renji Hospital Shanghai Jiao Tong University School of Medicine Shanghai Jiao Tong University Shanghai 200240 P. R. China; ^4^ Computational Biology group Department of Biomedical Engineering Eindhoven University of Technology The Netherlands; ^5^ Institute for Molecules and Materials Faculty of Science Radboud University Radboud University Heyendaalseweg 135 6525 AJ Nijmegen The Netherlands; ^6^ Center for Living Technologies, Alliance TU/e, WUR, UU, UMC Utrecht Princetonlaan 6 3584 CB Utrecht The Netherlands; ^7^ Department of Bionanoscience Kavli Institute of Nanoscience Delft University of Technology 2629 HZ Delft The Netherlands

**Keywords:** DNA, Enzymes, Molecular Communication, Synthetic Protocells

## Abstract

Protocells containing enzyme‐driven biomolecular circuits that can process and exchange information offer a promising approach for mimicking cellular features and developing molecular information platforms. Here, we employ synthetic transcriptional circuits together with CRISPR/Cas‐based DNA processing inside semipermeable protein‐polymer microcompartments. We first establish a transcriptional protocell that can be activated by external DNA strands and produce functional RNA aptamers. Subsequently, we engineer a transcriptional module to generate RNA strands functioning as diffusive signals that can be sensed by neighboring protocells and trigger the activation of internalized DNA probes or localization of Cas nucleases. Our results highlight the opportunities to combine CRISPR/Cas machinery and DNA nanotechnology for protocellular communication and provide a step towards the development of protocells capable of distributed molecular information processing.

## Graphical abstract

Engineering synthetic biochemical machinery for information processing and communication is essential for the construction of artificial protocells with complex dynamic behaviors. Here we use synthetic transcriptional circuits to produce diffusive RNA signal that can regulate CRISPR/Cas‐based DNA processing and localization in a binary community of synthetic protocells.

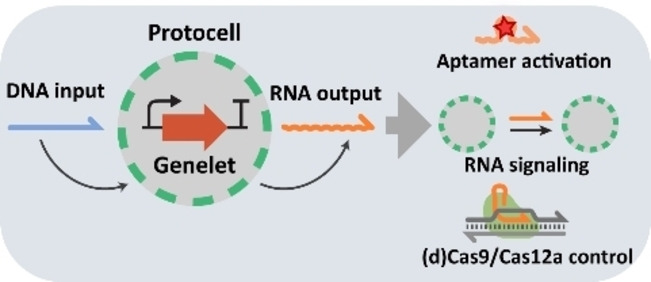

## Introduction

Biochemical reactions in living cells are connected and integrated by molecular communication to control collective behaviors.^[1]^ Inspired by nature, bottom‐up synthetic biology aims to decipher the governing principles of complex biological circuits and build programmable systems with new functionalities starting from molecular building blocks. A crucial step towards this goal is the creation and modulation of synthetic compartments consisting of minimalistic genetic biochemical machinery that performs information processing and decision making.[^2^, ^3^, ^4^, ^5^, ^6^, ^7^, ^8^, ^9^, ^10^] Non‐genetic biomolecular platforms can also be used for assembling reaction networks inside synthetic compartments. For example, enzymatic reactions have been used to engineer protocellular behaviors which include communication,[^11^, ^12^, ^13^] energy regeneration^[14]^ and prototissue contraction.^[15]^ While known for their robustness, such enzyme‐catalyzed pathways lack scalability towards advanced computational and decision‐making functions as they have relatively low orthogonality.

A promising alternative platform for bottom‐up construction of information processing and communication functionalities in synthetic protocells is DNA nanotechnology. The programmable and predictable nature of toehold‐mediated nucleic acid strand‐displacement (TMSD) reactions^[16]^ has been utilized to design a variety of dynamic molecular systems including logic circuits,[^17^, ^18^] neural networks,[^19^, ^20^] oscillators^[21]^ and gene expression classifiers.^[22]^ Recently, we employed TMSD circuits to construct a diffusion‐based communication platform capable of cascaded amplification, bidirectional signaling and computational operations in proteinosome‐based protocells.^[23]^ However, a limitation of systems that rely solely on TMSD is that nucleic acid strands cannot be produced or degraded and often complicated structures are required to mask the toeholds on unreacted species or waste products. Supplemented with enzymatic reactions, nucleic acid circuits can display a wider range of out‐of‐equilibrium behaviors while retaining their programmability and scalability.[^24^, ^25^, ^26^, ^27^] Simmel and co‐workers have investigated genelet‐based in vitro transcription (IVT) systems in compartmentalized artificial cell‐like environments and revealed the possibility of contact signaling.[^28^, ^29^] Because genelet modules produce RNA outputs, they could potentially be well suited for directing the activity of RNA‐guided CRISPR (clustered regularly interspaced short palindromic repeats)‐associated proteins (Cas). Cas nucleases have been repurposed as revolutionary tools in genome engineering,^[30]^ biosensors for diagnostics[^31^, ^32^] and DNA memory devices.^[33]^

**Figure 1:**
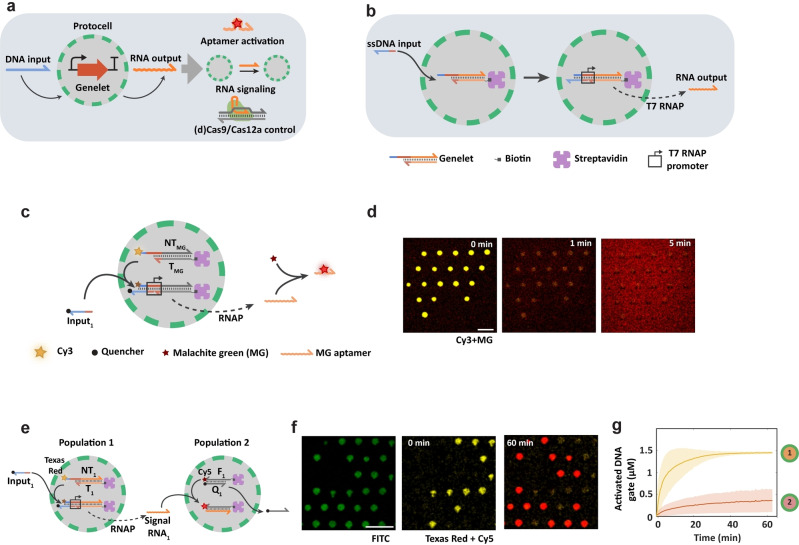
In vitro transcriptional modules in proteinosome‐based protocells. a) Schematic overview of a proteinosome‐localized transcriptional genelet module activated by a diffusive ssDNA input strand. Upon activation, the transcriptional switch produces RNA output strands which can perform multitude functionalities including aptamer formation, inter‐protocellular signaling or controlled activation of Cas nucleases. b) Molecular reaction diagram outlining the general design concept of the protocell‐encapsulated genelet. BSA‐NH_2_/PNIPAAm proteinosomes are assembled with 4 μM of internalized streptavidin, which has an encapsulation efficiency of around 25 %.^[23]^ The protocells have a size range of 10–60 μm in diameter (Figure S1). The template strand of the genelet duplex is localized in the proteinosomes using biotin‐streptavidin interaction. The genelet duplex contains an incomplete T7 RNAP promoter (red domain), which is completed upon binding the ssDNA input strand enabling RNA transcription by T7 RNAP. c) Schematic representation for the transcription of a malachite green (MG) aptamer in protocells. The domain **T_MG_NT_MG_** (grey, number of nucleotides, *n*=50 and 77, respectively) contains the sequence encoding the MG aptamer (*n*=38) which is transcribed upon binding of the input strand (**input_1_**, *n*=36). Localization of **input_1_** into protocells results in quenching of Cy3 fluorescence which is monitored using fluorescence microscopy. The transcribed MG aptamer binds MG which results in an increase in fluorescent intensity of MG. d) Confocal fluorescence micrographs of a population of MG‐genelet encapsulating proteinosomes in a microfluidic trapping array. At t=0 min, **input_1_** (500 nM), T7 RNAP (5 U/μL), MG (20 μM) and NTPs (3 mM each) were added to the trapping chamber, resulting in a fast decrease of the Cy3 fluorescence (yellow) in the proteinosomes. A gradual increase of MG fluorescence (red) occurs both within the protocells and in the external solution demonstrates successful transcription of the MG aptamer. Scale bar 100 μm. e) Schematic representation of an RNA signaling cascade between two proteinosome populations. Addition of **input_1_** triggers the activation of the transcriptional switch **T_1_NT_1_** (*n*=74 and 100, respectively) in population **1**, resulting in the transcription and secretion of an RNA signal strand. Simultaneously, binding of **input_1_** quenches the Texas Red fluorophore, thereby reporting switch activation. The secreted **RNA_1_** (*n*=62) signal diffuses through the external medium and is sensed by the DNA‐based **F_1_Q_1_**‐probe (*n*=50 and 36, respectively) localized in population **2**. RNA signal activates the probe via a TMSD reaction, resulting in displacement of quencher strand **Q_1_** and formation of a **RNA_1_**/**F_1_** duplex with a corresponding increase in Cy5 fluorescence. f) Confocal fluorescence micrographs of population **1** and **2** protocells (FITC‐labeled proteinosome membrane, green) showing time‐dependent decrease of Texas Red fluorescence (yellow) in population **1** and increase of Cy5 fluorescence (red) in population **2** associated with protocell activation. Scale bar 100 μm. g) Mean (solid line) and standard deviations (shaded areas) of the activated DNA concentrations of population **1** (yellow line) and population **2** (red line) in a microfluidic trapping device when **input_1_** (500 nM), T7 RNAP (5 U/μL) and NTPs (3 mM each) were added to the trapping chamber. The number of analyzed proteinosomes was 54. Fractions of the two populations were defined as q1 (population **1**) and q2 (population **2**), and q1=0.68 and q2=0.32, respectively. All experiments were performed at 37 °C.

Inspired by the high efficiency, specificity and programmability of enzyme‐driven DNA nanotechnology, herein we explore the potential of genelet IVT modules for implementing diffusive RNA signaling and information processing in semipermeable proteinosome‐based protocell communities (Figure 1a). As a compartment‐based molecular communication platform for integrating enzyme‐driven DNA nanotechnology into a CRISPR/Cas system is currently lacking, we sought to explore the potential of encapsulated transcriptional switches for the protocell‐mediated recruitment, localization and activation of Cas nucleases. To investigate how Cas nucleases could be integrated into protocellular networks, we demonstrate the selective encapsulation and activation of genelet templates within proteinosomes, which upon addition of a membrane‐permeable single‐stranded DNA (ssDNA) input triggers the synthesis of a single‐stranded RNA output (Figure 1a). We first show that the produced RNA output functions as a malachite green aptamer, indicating that the protocell‐entrapped genelet is functional and accessible to nucleoside triphosphates (NTPs) and T7 RNA polymerase (RNAP) present in the external solution. Subsequently, we establish diffusive RNA‐based signaling between protocells in a microfluidic trapping array and investigate strategies for triggering the activities of Cas12a and (d)Cas9 nucleases in protocells. Finally, we successfully implement genelet controlled localization and activation of dCas9 in binary populations of protocells. Taken together, our results highlight the opportunities for combining the programmability of transcriptional switches with the capabilities of Cas nucleases in protocellular signaling networks and provide a step towards the development of protocell platforms based on distributed molecular sensing and processing.

## Results and Discussion

### In Vitro Transcriptional Modules in Proteinosome‐based Protocells

In previous work, we demonstrated that proteinosomes, internally hollow microcapsules constructed from bovine serum albumin/poly(*N*‐isopropylacrylamide) (BSA‐NH_2_/PNIPAAm) nanoconjugates,[^23^, ^34^] are permeable to double‐stranded DNA (dsDNA) oligonucleotides of up to 100 base pairs (bp) (ca. 65 kDa) through passive diffusion (Figure S1) and encapsulated streptavidin allows for stable localization of biotin‐functionalized DNA.^[23]^ Thus, to enable RNA transcription specifically within the proteinosomes, we functionalized the template strand of a genelet duplex with biotin such that the transcriptional switch was spatially confined within streptavidin‐containing proteinosomes (Figure 1b, Materials and methods). The genelet IVT module contains an incomplete T7 RNA polymerase (RNAP) promoter region, from which transcription is severely inhibited and showing low backgrounds.^[35]^ To activate the transcriptional switch, we designed an ssDNA input strand that hybridizes with the single stranded region of the non‐template strand of the genelet duplex, thereby completing the RNAP promoter region. To verify that the protocell‐encapsulated genelet is functional and T7 RNAP (ca. 100 kDa) and NTPs can permeate the proteinosome membrane to initiate RNA transcription, we used a genelet that transcribes the malachite green (MG) aptamer sequence (Figure 1c). PAGE analysis showed the production of MG aptamer, which binds to MG fluorophore and thereby strongly increases its fluorescence (Figure S2).^[36]^ To observe the hybridization of the ssDNA input strand **input_1_** with the non‐template strand **NT_MG_** of the genelet (template strand **T_MG_**), a Cy3 fluorophore was added to the 5′ end of **NT_MG_** and a quencher to the 3′ end of **input_1_** strand. A decrease in Cy3 fluorescence inside the protocells is indicative of **input_1_** binding.

We first confirmed successful permeation and accumulation of Cy5 fluorophore labelled T7 RNAP into proteinosomes with localized genelet target using confocal microscopy (Figure S3). We then localized a population of genelet‐containing proteinosomes in a microfluidic trapping array^[23]^ and subsequently flowed **input_1_**, T7 RNAP and NTPs into the chamber (Materials and methods). Fluorescence data revealed a decrease of Cy3 fluorescence (yellow) inside the proteinosomes immediately after the addition of the **input_1_** and a gradual increase of MG fluorescence (red) in the areas surrounding the proteinosomes (Figure 1d). In the absence of **input_1_** or T7 RNAP, no increase in malachite green fluorescence was observed (Figure S4). These results showed that the proteinosome‐encapsulated genelet is functional and T7 RNAP is able to enter the protocells and transcribe RNA strands containing the MG aptamer sequence.

Transmission of soluble signaling molecules between different cell types is a key characteristic of multicellular organisms, triggering and regulating processes such as growth, differentiation and gene expression.^[37]^ Having established a functional transcriptional switch in protocells, we proceeded to investigate whether the transcribed RNA strands could function as diffusive signaling molecules capable of activating other protocells. We designed an RNA based protocellular signaling system with two proteinosome populations (Figure 1e). Population **1** contains a genelet **T_1_NT_1_** consisting of a Texas Red‐labeled strand **NT_1_** and biotinylated strand **T_1_**, which transcribes a diffusible RNA signal **RNA_1_** upon input binding. Population **2** contains a streptavidin‐anchored TMSD DNA probe with a fluorophore‐quencher pair **F_1_Q_1_** probe where the Cy5‐modified top strand (**F_1_**) has a sequence that is fully complementary to signal **RNA_1_**. Addition of quencher‐labeled **input_1_** results in a decrease in Texas Red fluorescence and initiates transcription in population **1**, thereby triggering TMSD reactions in population **2**. Batch measurements revealed that the Cy5 fluorescence of the **F_1_Q_1_** probe showed a significant increase only in the presence of the genelet and input (Figure S5). According to previous study,^[38]^ the amount of DNA template proportionally increases the production of RNA transcription when the template is limiting and the transcription plateaus around 1.25 μM of DNA template. Therefore, we used an internalized genelet with a concentration around 1–1.5 μM, which was in the range of maximum RNA production. The two populations were trapped in the microfluidic device and their fluorescence levels were measured. Initially, population **1** displayed high levels of Texas Red fluorescence (yellow) and population **2** showed low levels of Cy5 fluorescence (red), corresponding to inactive state of the genelet and quenched state of the **F_1_Q_1_** probe respectively (Figure 1f). When the ssDNA **input_1_**, T7 RNAP and NTPs were loaded into the trapping chamber, population **1** fluorescence dropped, indicating activation of the genelet. In contrast, the fluorescence of population **2** increased, corresponding to the activation of the **F_1_Q_1_** probe and the formation of the RNA/DNA hybrid duplex. To analyze the activation dynamics we plotted the average fluorescence traces (Figure 1g). Without **input_1_**, only very low levels of **F_1_Q_1_** probe activation in population **2** was detected (Figure S6), possibly because low levels of leaky transcription can still occur from the incomplete promoter.^[24]^ Additionally, we added **RNA_1_** to protocells containing **F_1_Q_1_** or scrambled **F_s_Q_s_** probes. The data showed only **F_1_Q_1_**‐containing protocells were activated, indicating target specificity (Figure S7). The results establish that the RNA strands produced from the protocell‐encapsulated genelet can execute diffusive molecular communication between two protocell populations through TMSD reactions.

### Guiding Cas Nucleases to Proteinosome‐localized DNA Targets

**Figure 2:**
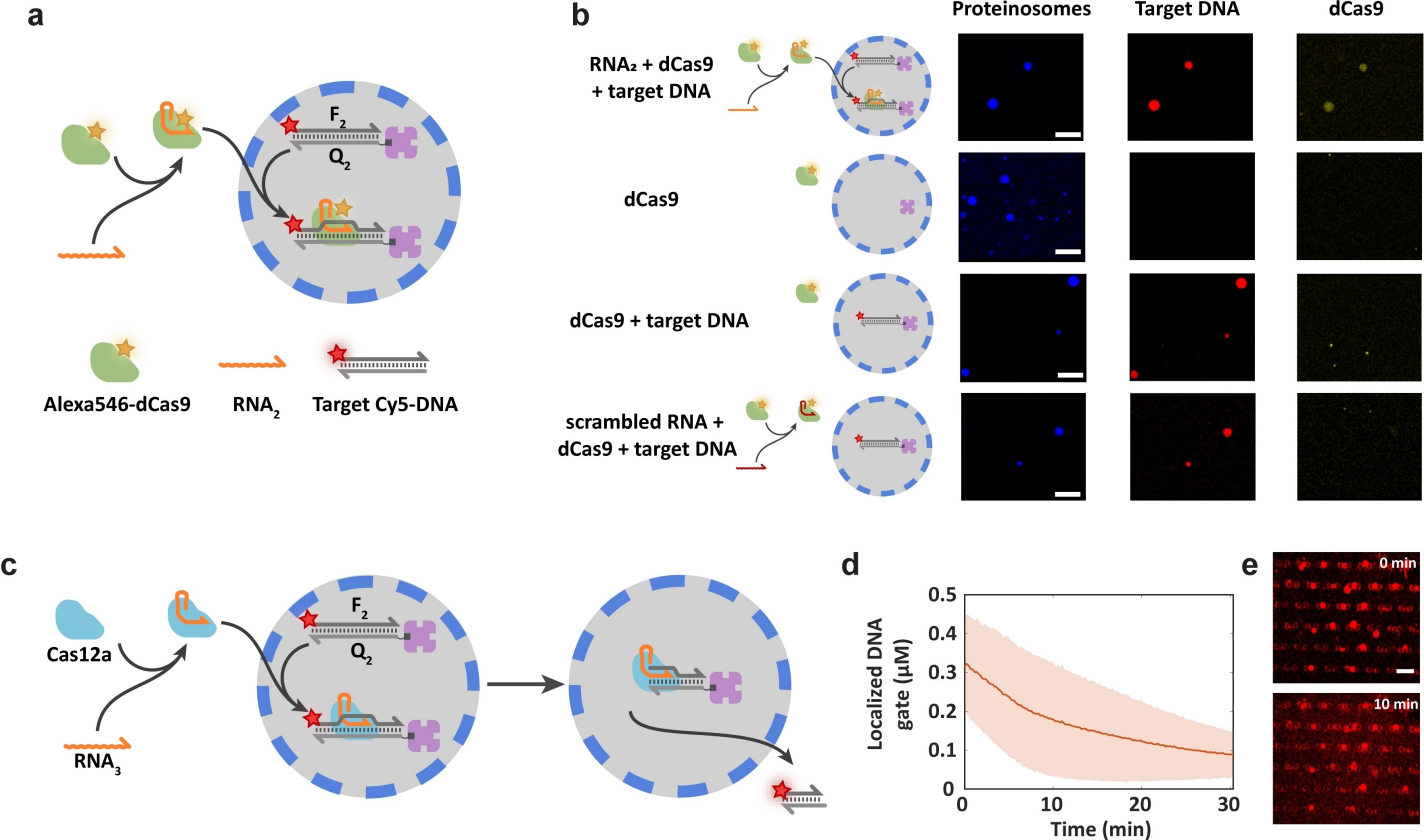
Guiding Cas nucleases to proteinosome‐localized DNA targets. a) Schematic representation of dCas9 localization strategy. Protocells that contain a Cy5‐labeled 190mer dsDNA target **F_2_Q_2_** (190 bp) with a dCas9 PAM sequence and a biotin modification were encapsulated in streptavidin‐containing proteinosomes. The dCas9 was labeled with Alexa546 to observe the binding with internalized DNA target in the presence of single guide RNA (**RNA_2_**, *n*=101). b) Confocal fluorescence micrographs of Dylight405‐labeled proteinosomes (blue) that contain a dsDNA target (red) after 15 mins incubation with Alexa546‐dCas9 (yellow) (500 nM) and sgRNA strand **RNA_2_** (1 μM) demonstrates localization of dCas9 (top row). Control experiments using empty protocells (second row) or protocells that contain the dsDNA target but with no sgRNA addition (third row) or scrambled sgRNA (*n*=100) (bottom row) do not show localization of dCas9, indicating target specificity. Scale bars 100 μm. c) Schematic representation of Cas12a‐based cleavage and release of proteinosome‐localized dsDNA targets. Proteinosomes with a Cy5‐labeled dsDNA target **F_2_Q_2_** are trapped in a microfluidic device and target cleavage is initiated by addition of Cas12a and **RNA_3_** (*n*=40) d) Mean and standard deviation of the concentrations of DNA target **F_2_Q_2_** in a population of proteinosomes after addition of Cas12a (1 μM) and sgRNA **RNA_3_** (1 μM) to the trapping chamber. The number of analyzed proteinosomes was 39. e) Confocal fluorescence micrographs corresponding to the data in (d) at time points immediately before cleavage and 10 mins after cleavage. The increased Cy5 fluorescence in the external medium in the bottom image indicates the presence of cleaved DNA targets that are diffused out of the protocells. Scale bar 100 μm. All experiments were performed at 37 °C.

Given that Cas nucleases can be controlled with a single guide RNA (sgRNA) strand for a wide range of applications,^[30]^ we sought to use sgRNA as a signal for recruiting Cas nucleases into proteinosome‐based protocells containing localized dsDNA targets. To verify that CRISPR/Cas technology is compatible with proteinosome‐based protocells, we used dCas9 (catalytically dead Cas9) as a model Cas protein and labeled it with an Alexa546 fluorophore (Materials and methods). Fluorescence microscopy was used to observe dCas9‐sgRNA (**RNA_2_**) complex uptake into proteinosomes containing entrapped dsDNA targets **F_2_Q_2_** consisting of a dCas9 PAM target sequence, Cy5 fluorophore and biotin modifications (Figure 2a). Initially, we observed that proteinosomes prepared using a typical concentration of the membrane nanoconjugate^[23]^ (BSA/PNIPAAm concentration, 16 mg mL^−1^) were less permeable to 190mer dsDNA (ca. 120 kDa), such that loadings of the target DNA were minimal (Figure S8). Experiments were therefore undertaken using proteinosomes prepared at lower concentration of nanoconjugate (8 mg mL^−1^), which were more permeable to the target dsDNA. Under these conditions, confocal fluorescence micrographs showed that dCas9‐Alexa546 is significantly localized into the DNA target‐containing proteinosomes (Figure 2b, top row and Figure S9). dCas9 localization was heterogeneous, possibly resulting from the relatively large molecular weight of dCas9 (ca. 160 kDa) and great variability in proteinosome permeabilities. Only minimal levels of dCas9‐Alexa546 localization within empty proteinosomes is detected (Figure 2b, second row). Experiments without sgRNA or with a scrambled sgRNA showed a similarly low level of non‐specific adsorption (Figure 2b, third and bottom rows). These results indicate that dCas9 localizes in dsDNA target‐containing proteinosomes in the presence of the cognate sgRNA.

As Cas nucleases are used in genome engineering to create a double‐stranded break at a location specified by a sgRNA which is then repaired by cellular DNA repair pathways that introduce small deletions, insertions or a donor dsDNA sequence into the site of the break,^[39]^ we investigated whether Cas‐based cleavage reactions could be implemented within synthetic protocells. Under in vitro conditions, Cas9 remains tightly bound to both ends of the cleaved DNA target with lifetimes of over 5 h.^[40]^ Therefore, to demonstrate protocell‐located Cas activity, we used Cas12a (Cpf1), which immediately releases the PAM‐distal end of the cleaved DNA target.^[41]^ The Cy5‐labeled dsDNA target **F_2_Q_2_** with a Cas12a PAM target sequence was localized in proteinosomes through streptavidin‐biotin interactions (Figure 2c). Fluorescence measurements showed a decrease in proteinosome‐localized Cy5 fluorescence when Cas12a and sgRNA (**RNA_3_**) were added to the external medium (Figure 2d). Additionally, an increase of Cy5 fluorescence in the external medium was clearly visible from confocal fluorescence images (Figure 2e). A control experiment was performed without **RNA_3_** showing no decrease in Cy5 fluorescence (Figure S10). Together, these results validate that cleavage of proteinosome‐encapsulated dsDNA can be achieved using Cas12a and the cognate sgRNA.

### Genelet‐triggered Activation of dCas9 Nuclease in Protocell Communities

**Figure 3:**
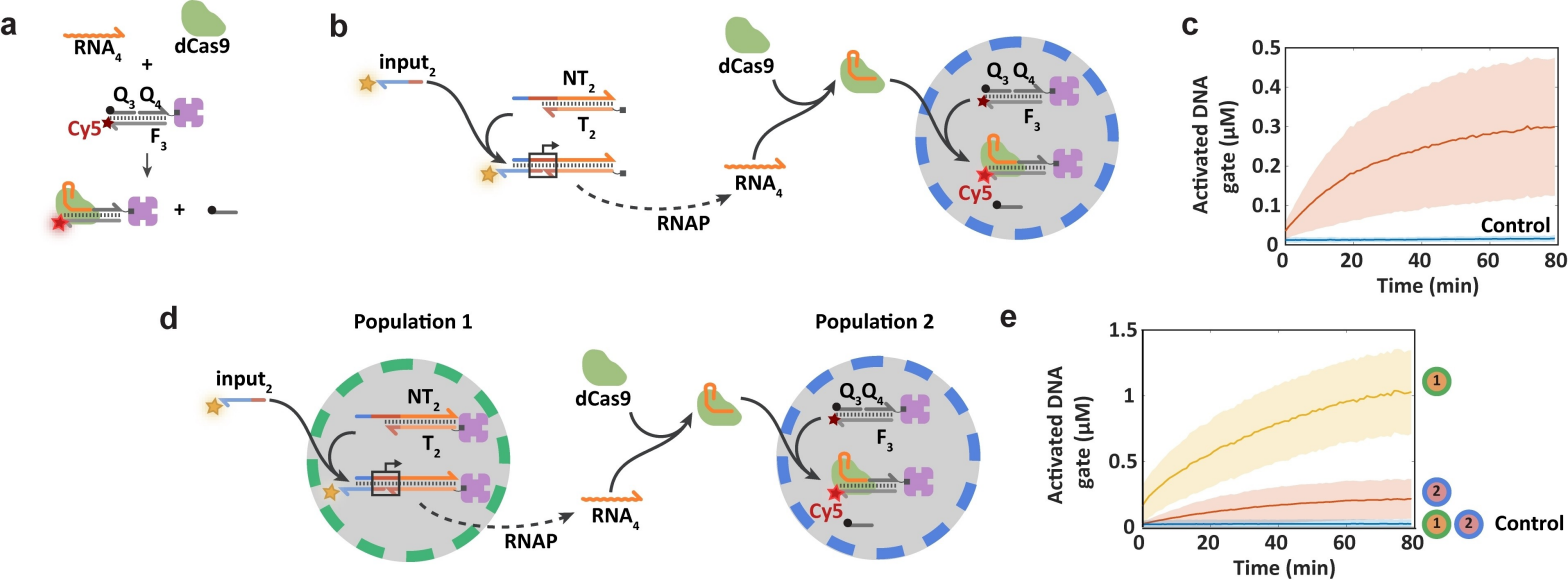
Genelet‐triggered activation of dCas9 nuclease in protocell communities. a) Schematic representation of the dCas9‐localizing fluorescent probe. The probe consists of a nicked DNA duplex (**F_3_Q_3_Q_4_**, *n*=50, 23, 27, respectively) with a Cy5 FQ pair where the short quencher strand is displaced upon binding of the dCas9‐sgRNA (**RNA_4_**, *n*=100) complex thereby increasing Cy5 fluorescence. b) Schematic representation of the strategy for genelet controlled dCas9 targeting into proteinosomes under batch conditions. A genelet duplex **T_2_NT_2_** (*n*=113 and 138, respectively) with an output domain encoding the sequence of **RNA_4_** is added to a microfluidic chamber containing trapped proteinosomes with encapsulated Cy5‐labeled dCas9‐localizing probes. Transcription of **RNA_4_** is initiated by the addition of a Cy3‐labeled ssDNA **input_2_** (*n*=36) resulting in the formation of a dCas9‐sgRNA complex and binding to the proteinosome‐localized fluorescent probe. c) Mean and standard deviation of the concentrations of the activated dCas9 probe in proteinosomes (red line) when the genelet **T_2_NT_2_** (0.5 μM), **input_2_** (0.5 μM), T7 RNAP (20 U/μL), dCas9 (1 μM) and NTPs (3 mM each) were added to the trapping chamber. As a control experiment, the concentration of activated dCas9 probe in proteinosomes without DNA **input_2_** is also plotted (blue line). The numbers of analyzed proteinosomes in the presence and absence of input were 42 and 53, respectively. d) Schematic representation of the strategy for compartmentalized genelet controlled proteinosome‐localized dCas9 activation. A genelet duplex **T_2_NT_2_** with an output domain encoding the sequence of **RNA_4_** is localized in proteinosome population **1** while population **2** contains the dCas9‐localizing fluorescent probe. Transcription of the **RNA_4_** signal strand in population **1** is initiated upon the addition of an ssDNA input strand (**input_2_**). The RNA signal is free to diffuse to population **2** and triggers the formation of a dCas9‐sgRNA complex, resulting in activation of the proteinosome‐localized Cy5 fluorescent probe. e) Mean and standard deviation of the concentrations of activated genelet **T_2_NT_2_** in population **1** (yellow line) and activated dCas9 probe in population **2** (red line) when **input_2_** (0.5 μM), T7 RNAP (20 U/μL), dCas9 (1 μM) and NTPs (3 mM each) were added to the trapping chamber. A control experiment was performed in the absence of **input_2_** showing no activation in both population **1** and **2** (blue lines). The numbers of analyzed proteinosomes in the presence and absence of input were 88 and 72, respectively. Fractions of the two populations in the presence and absence of input were q1=0.73, q2=0.27 and q1=0.72, q2=0.28, respectively. In the experiment with DNA **input_2_**, a small number of protocells that were impermeable to dCas9 (Figure S9) were excluded from analysis (Figure S15). All experiments were performed at 37 °C.

Given that recruitment and activation of Cas12a within DNA target‐containing proteinosomes can be achieved by adding an appropriately designed RNA strand, we sought to replace the extraneous input with a triggerable RNA signal derived endogenously from genelet‐containing proteinosomes to produce binary protocell networks capable of programmable Cas recruitment and activation. To achieve this, we used tightly bound (d)Cas9 in place of Cas12a, as the latter can exhibit indiscriminate DNA nuclease activity after target binding,^[42]^ which in principle could unselectively degrade the circuit components after activation in protocell‐based DNA circuits. In addition, based on a fluorescent (d)Cas9 beacon,^[43]^ we assembled a nicked dsDNA target with an FQ (fluorophore‐quencher) pair and a biotin modification on opposite ends (Figure 3a). In this three‐strand complex **F_3_Q_3_Q_4_**, the nick is just adjacent to the PAM‐sequence, allowing the quencher‐bearing single strand **Q_3_** to be displaced by the (d)Cas9‐sgRNA complex and released from the beacon, which could be monitored by an increase in Cy5 fluorescence. Functionalities of the (d)Cas9 probes were first confirmed in batch conditions (Figure S11), which showed that activation was observed only if both (d)Cas9 and the corresponding sgRNA were present. The nicked **F_3_Q_3_Q_4_** probe was then localized in a population of proteinosomes and analyzed in a microfluidic trapping array. Fluorescence measurements of the proteinosomes population revealed that the encapsulated probe was rapidly activated when dCas9 and the corresponding sgRNA strand (**RNA_4_**) were added to the trapping chamber (Figure S12). If required, the nicked **F_3_Q_3_Q_4_** probe can also be activated using Cas9 (Figure S13). However, while Cas9 is also expected to cleave the Cy5‐labeled strand, this is not detected under in vitro conditions as Cas9 stays tightly bound to both ends of the target dsDNA.^[40]^ Together, these results confirm that the nicked **F_3_Q_3_Q_4_** probe can be used to monitor the activity of dCas9 nuclease and generate an ssDNA as output in proteinosome‐based protocells.

Given the above observations, we designed a genelet module **T_2_NT_2_** that upon activation by an ssDNA input (**input_2_**) transcribes sgRNA strand **RNA_4_** that would trigger the localization of dCas9 into proteinosomes containing the localized nicked **F_3_Q_3_Q_4_** probe (Figure 3b). The performance of this circuit was verified in batch conditions, confirming that significant dCas9 binding to the fluorescent target dsDNA occurs in the presence of the initial ssDNA input, genelet duplex, sgRNA, T7 RNAP and NTPs (Figure S14). A population of proteinosomes with encapsulated fluorescent dCas9 probe was then trapped in a microfluidic device and fluorescence data showed the activation of dCas9 probe after the addition of the active genelet module, T7 RNAP and NTPs (Figure 3c, red line). As expected, the activation using the free genelet was slower compared to the direct addition of the sgRNA (Figure S12). Further, a control experiment using an inactive genelet module, T7 RNAP and NTPs showed minimal dCas9 activation (Figure 3c, blue line).

Finally, we implemented the above circuit within a network comprising two populations of protocells. For this, we assembled a dCas9 activation system triggered by genelet‐containing proteinosomes in binary populations trapped in the microfluidic device (Figure 3d). Population **1** contains a genelet **T_2_NT_2_** that transcribes the diffusible sgRNA signal (**RNA_4_**) upon addition of ssDNA **input_2_**. Population **2** contains the nicked **F_3_Q_3_Q_4_** probe. The experiment was initiated by addition of ssDNA input strand **input_2_**, T7 RNAP and NTPs to the device resulting in the completion of genelet promotor region which could be monitored by an increase in Cy3 fluorescence (Figure 3e, yellow line). The generated **RNA_4_** was released from population **1** and freely diffuses into the external environment resulting in the formation of dCas9‐sgRNA complex and subsequent activation of population **2**, as shown by an increase in Cy5 fluorescence (Figure 3e, red line). A control experiment in the absence of **input_2_** displayed no activation in both population **1** and **2** (Figure 3e, blue lines). Collectively, these results show that genelet transcriptional switches are able to trigger dCas9 activation within distributed compartmentalized DNA circuits.

## Conclusion

In this work, we demonstrate how synthetic transcriptional switches regulate Cas nucleases in proteinosome‐based compartmentalized DNA circuits through diffusive molecular communication. In general, we use proteinosomes rather than lipid vesicles because high molecular weight biomolecules such as T7 RNAP and Cas nucleases can access the water‐filled interior after assembly of the protocells, thereby circumventing potential problems associated with loss of function during proteinosome preparation and storage. Post‐assembly entrapment of information processing modules such as genelet duplexes and TMSD probes is achieved by anchoring to pre‐encapsulated streptavidin. Using this strategy, we show that protocell‐encapsulated genelet transcriptional switches are used to secrete diffusible RNA strands. Compared to DNA‐based diffusible signals,[^23^, ^44^] RNA strands have a more diverse functional repertoire including folding into an aptamer and activation of a DNA‐based probe located in a different protocell population. Our results highlight the potential for constructing new artificial cells networks by taking advantage of enzymatically driven RNA production and integration of RNA outputs with CRISPR/Cas modules.

Finally, we use diffusible RNA signals to execute the binding of Cas12a and (d)Cas9 to proteinosome‐encapsulated DNA targets located within communities of protocells. Cas12a releases the PAM‐proximal end of the cleaved target, but it also exhibits indiscriminate DNase activity, which can limit possible applications in distributed DNA‐based circuits. In contrast, transcriptional protocell‐mediated recruitment of dCas9 into proteinosomes containing an encapsulated nicked dsDNA probe results in binding of dCas9 and release of a single‐stranded output. The resulting output can potentially be used as diffusive signal for constructing more complex protocell communication networks. To realize such a system, the kinetic mechanism of strand displacement from a nicked dsDNA mediated by dCas9/sgRNA complex first needs to be investigated in detail. A limitation of our methodology is the heterogeneity of dCas9 diffusion and activation in protocells due to its large protein size, which in principle can be circumvented by using smaller Cas proteins developed recently.^[45]^ Additionally, the (d)Cas9 used in our system is constrained by the very slow target release rates, which could possibly be mitigated using recently discovered methods that permit (d)Cas9 multi‐turnover behavior in vitro.^[46]^ Because of the high orthogonality of RNA‐based communication channels, compartmentalized CRISPR/Cas‐based DNA/RNA circuits are highly scalable, which would extend the capability of multiplexing in Cas‐based diagnostic tools. For example, the CRISPR‐based nucleic acid detection assay SHERLOCK contains two steps, namely the pre‐amplification of a DNA/RNA input to RNA via T7 transcription and the subsequent detection by a Cas/sgRNA complex. These processes can be seamlessly implemented in our system for simultaneous testing of multiple targets via distributed signal sensing and processing.^[47]^ Furthermore, our system opens new possibilities to employ a wide range of CRISPR‐Cas‐derived editing agents including base editors^[48]^ capable of recording cellular history by producing single‐base modifications within protocells. Design and construction of protocells with writable memory via CRISPR editing tools for use in data storage will be investigated in future work.

## Conflict of interest

The authors declare no conflict of interest.

1

## Supporting information

As a service to our authors and readers, this journal provides supporting information supplied by the authors. Such materials are peer reviewed and may be re‐organized for online delivery, but are not copy‐edited or typeset. Technical support issues arising from supporting information (other than missing files) should be addressed to the authors.

Supporting Information

## Data Availability

The data that support the findings of this study are available from the corresponding author upon reasonable request.

## References

[1] J. A. Papin , T. Hunter , B. O. Palsson , S. Subramaniam , Nat. Rev. Mol. Cell Biol. 2005, 6, 99–111. 15654321 10.1038/nrm1570

[2] P. Van Nies , I. Westerlaken , D. Blanken , M. Salas , M. Mencía , C. Danelon , Nat. Commun. 2018, 9, 1583. 29679002 10.1038/s41467-018-03926-1 PMC5910420

[3] Y. Elani , R. V. Law , O. Ces , Phys. Chem. Chem. Phys. 2015, 17, 15534–15537. 25932977 10.1039/c4cp05933f

[4] T. Y. D. Tang , D. Cecchi , G. Fracasso , D. Accardi , A. Coutable-Pennarun , S. S. Mansy , A. W. Perriman , J. L. R. Anderson , S. Mann , ACS Synth. Biol. 2018, 7, 339–346. 29091420 10.1021/acssynbio.7b00306

[5] K. P. Adamala , D. A. Martin-Alarcon , K. R. Guthrie-Honea , E. S. Boyden , Nat. Chem. 2017, 9, 431–439. 28430194 10.1038/nchem.2644 PMC5407321

[6] E. Godino , J. N. López , D. Foschepoth , C. Cleij , A. Doerr , C. F. Castellà , C. Danelon , Nat. Commun. 2019, 10, 4969. 31672986 10.1038/s41467-019-12932-w PMC6823393

[7] H. Niederholtmeyer , C. Chaggan , N. K. Devaraj , Nat. Commun. 2018, 9, 5027. 30487584 10.1038/s41467-018-07473-7 PMC6261949

[8] K. A. Ganzinger , P. Schwille , J. Cell Sci. 2019, 132, jcs227488. 30718262 10.1242/jcs.227488

[9] J. C. Blain , J. W. Szostak , Annu. Rev. Biochem. 2014, 83, 615–640. 24606140 10.1146/annurev-biochem-080411-124036

[10] N. A. Yewdall , A. F. Mason , J. C. M. Van Hest , Interface Focus 2018, 8, 20180023. 30443324 10.1098/rsfs.2018.0023 PMC6227776

[11] A. F. Mason , B. C. Buddingh’ , D. S. Williams , J. C. M. van Hest , J. Am. Chem. Soc. 2017, 139, 17309–17312. 29134798 10.1021/jacs.7b10846 PMC5724030

[12] Y. Elani , R. V. Law , O. Ces , Nat. Commun. 2014, 5, 5305. 25351716 10.1038/ncomms6305

[13] B. C. Buddingh , J. Elzinga , J. C. M. Van Hest , Nat. Commun. 2020, 11, 1652. 32246068 10.1038/s41467-020-15482-8 PMC7125153

[14] K. Y. Lee , S.-J. Park , K. A. Lee , S.-H. Kim , H. Kim , Y. Meroz , L. Mahadevan , K.-H. Jung , T. K. Ahn , K. K. Parker , Nat. Biotechnol. 2018, 36, 530–535. 29806849 10.1038/nbt.4140

[15] P. Gobbo , A. J. Patil , M. Li , R. Harniman , W. H. Briscoe , S. Mann , Nat. Mater. 2018, 17, 1145–1153. 30297813 10.1038/s41563-018-0183-5

[16] D. Y. Zhang , E. Winfree , J. Am. Chem. Soc. 2009, 131, 17303–17314. 19894722 10.1021/ja906987s

[17] G. Seelig , D. Soloveichik , D. Y. Zhang , E. Winfree , Science 2006, 314, 1585–1588. 17158324 10.1126/science.1132493

[18] L. Qian , E. Winfree , Science 2011, 332, 1196–1201. 21636773 10.1126/science.1200520

[19] K. M. Cherry , L. Qian , Nature 2018, 559, 370–376. 29973727 10.1038/s41586-018-0289-6

[20] L. Qian , E. Winfree , J. Bruck , Nature 2011, 475, 368–372. 21776082 10.1038/nature10262

[21] N. Srinivas , J. Parkin , G. Seelig , E. Winfree , D. Soloveichik , Science 2017, 358, eaal2052. 29242317 10.1126/science.aal2052

[22] R. Lopez , R. Wang , G. Seelig , Nat. Chem. 2018, 10, 746–754. 29713032 10.1038/s41557-018-0056-1

[23] A. Joesaar , S. Yang , B. Bögels , A. van der Linden , P. Pieters , B. P. Kumar , N. Dalchau , A. Phillips , S. Mann , T. F. A. de Greef , Nat. Nanotechnol. 2019, 14, 369–378. 30833694 10.1038/s41565-019-0399-9 PMC6451639

[24] J. Kim , K. S. White , E. Winfree , Mol. Syst. Biol. 2006, 2, 68. 17170763 10.1038/msb4100099 PMC1762086

[25] K. Montagne , R. Plasson , Y. Sakai , T. Fujii , Y. Rondelez , Mol. Syst. Biol. 2011, 7, 466. 21283142 10.1038/msb.2010.120 PMC3063689

[26] A. Padirac , T. Fujii , Y. Rondelez , Proc. Natl. Acad. Sci. USA 2012, 109, E3212–E3220. 23112180 10.1073/pnas.1212069109 PMC3511151

[27] J. Kim , E. Winfree , Mol. Syst. Biol. 2011, 7, 465. 21283141 10.1038/msb.2010.119 PMC3063688

[28] M. Weitz , J. Kim , K. Kapsner , E. Winfree , E. Franco , F. C. Simmel , Nat. Chem. 2014, 6, 295–302. 24651195 10.1038/nchem.1869

[29] A. Dupin , F. C. Simmel , Nat. Chem. 2019, 11, 32–39. 30478365 10.1038/s41557-018-0174-9 PMC6298583

[30] G. J. Knott , J. A. Doudna , Science 2018, 361, 866–869. 30166482 10.1126/science.aat5011 PMC6455913

[31] J. S. Gootenberg , O. O. Abudayyeh , M. J. Kellner , J. Joung , J. J. Collins , F. Zhang , Science 2018, 360, 439–444. 29449508 10.1126/science.aaq0179 PMC5961727

[32] J. S. Gootenberg , O. O. Abudayyeh , J. W. Lee , P. Essletzbichler , A. J. Dy , J. Joung , V. Verdine , N. Donghia , N. M. Daringer , C. A. Freije , Science 2017, 356, 438–442. 28408723 10.1126/science.aam9321 PMC5526198

[33] R. U. Sheth , H. H. Wang , Nat. Rev. Genet. 2018, 19, 718–732. 30237447 10.1038/s41576-018-0052-8 PMC6492567

[34] X. Huang , M. Li , D. C. Green , D. S. Williams , A. J. Patil , S. Mann , Nat. Commun. 2013, 4, 2239. 23896993 10.1038/ncomms3239

[35] C. T. Martin , J. E. Coleman , Biochemistry 1987, 26, 2690–2696. 3300768 10.1021/bi00384a006

[36] D. Grate , C. Wilson , Proc. Natl. Acad. Sci. USA 1999, 96, 6131–6136. 10339553 10.1073/pnas.96.11.6131 PMC26847

[37] B. A. Doğaner , L. K. Q. Yan , H. Youk , Trends Cell Biol. 2016, 26, 262–271. 26671200 10.1016/j.tcb.2015.11.002

[38] Z. J. Kartje , H. I. Janis , S. Mukhopadhyay , K. T. Gagnon , J. Biol. Chem. 2021, 296, 100175. 33303627 10.1074/jbc.RA120.014553 PMC7948468

[39] K. S. Pawelczak , N. S. Gavande , P. S. VanderVere-Carozza , J. J. Turchi , ACS Chem. Biol. 2018, 13, 389–396. 29210569 10.1021/acschembio.7b00777

[40] C. D. Richardson , G. J. Ray , M. A. DeWitt , G. L. Curie , J. E. Corn , Nat. Biotechnol. 2016, 34, 339–344. 26789497 10.1038/nbt.3481

[41] D. Singh , J. Mallon , A. Poddar , Y. Wang , R. Tippana , O. Yang , S. Bailey , T. Ha , Proc. Natl. Acad. Sci. USA 2018, 115, 5444–5449. 29735714 10.1073/pnas.1718686115 PMC6003496

[42] J. S. Chen , E. Ma , L. B. Harrington , M. Da Costa , X. Tian , J. M. Palefsky , J. A. Doudna , Science 2018, 360, 436–439. 29449511 10.1126/science.aar6245 PMC6628903

[43] V. Mekler , L. Minakhin , E. Semenova , K. Kuznedelov , K. Severinov , Nucleic Acids Res. 2016, 44, 2837–2845. 26945042 10.1093/nar/gkw138 PMC4824121

[44] S. Yang , P. A. Pieters , A. Joesaar , B. W. A. Bögels , R. Brouwers , I. Myrgorodska , S. Mann , T. F. A. de Greef , ACS Nano 2020, 14, 15992–16002. 33078948 10.1021/acsnano.0c07537 PMC7690052

[45] X. Xu , A. Chemparathy , L. Zeng , H. R. Kempton , S. Shang , M. Nakamura , L. S. Qi , Mol. Cell 2021, 81, 4333–4345. 34480847 10.1016/j.molcel.2021.08.008

[46] A. S. Wang , L. C. Chen , R. A. Wu , Y. Hao , D. T. McSwiggen , A. B. Heckert , C. D. Richardson , B. G. Gowen , K. R. Kazane , J. T. Vu , Mol. Cell 2020, 79, 221–233. 32603710 10.1016/j.molcel.2020.06.014 PMC7398558

[47] M. M. Kaminski , O. O. Abudayyeh , J. S. Gootenberg , F. Zhang , J. J. Collins , Nat. Biomed. Eng. 2021, 5, 643–656. 34272525 10.1038/s41551-021-00760-7

[48] A. V. Anzalone , L. W. Koblan , D. R. Liu , Nat. Biotechnol. 2020, 38, 824–844. 32572269 10.1038/s41587-020-0561-9

