# Supplementary material for: Protocellular CRISPR/Cas‐Based Diffusive Communication Using Transcriptional RNA Signaling
Source: Angew Chem Int Ed Engl. 2022 Apr 26;61(26):e202202436. doi: 10.1002/anie.202202436 (PMC9320857; doi:10.1002/anie.202202436)
Supplement: Supplementary file 1 — Supporting Information [file ANIE-61-0-s001.pdf]

Angewandte Chemie
Eine Zeitschrift der Gesellschaft Deutscher Chemiker

Supporting Information

Protocellular CRISPR/Cas-Based Diffusive Communication Using Transcriptional RNA Signaling

S. Yang, A. Joesaar, B. W. A. Bögels, S. Mann, T. F. A. de Greef**

Supporting Information

Supplementary Methods

Materials

2-Ethyl-1-hexanol (Sigma, 98%), 1-(3-dimethylaminopropyl)-3-ethylcarbodiimide HCl (EDC, Carbosynth), 1,6-diaminohexane (Sigma, 98%), Fluorescein isothiocyanate (FITC, 90%, Sigma), PEG-bis(N-succinimidyl succinate) (Mw = 2000, Sigma), streptavidin from Streptomyces avidinii (Sigma), DyLightTM 405 NHS ester (ThermoFisher), Sulfo-Cy5 NHS ester (ThermoFisher), streptavidin, Alexa FluorTM 546 conjugate (ThermoFisher), bovine serum albumin (heat shock fraction, pH 7, 98%, Sigma), EvaGreen (Biotium), SYBR Gold (ThermoFisher), GeneRuler DNA ladder (ThermoFisher), KAPA HiFi Hot- Start Polymerase (KAPA Biosystems), EnGenTM sgRNA Synthesis Kit, S. pyogenes (NEB), MonarchTM RNA Cleanup Kit (50 µg) (NEB), SNAP-SurfaceTM Alexa FluorTM 546 (NEB), EnGenTM Lba Cas12a (Cpf1) (NEB), Cas9 Nuclease, S. pyogenes (NEB), EnGenTM Spy dCas9 (SNAP-tagTM) (NEB), T7 RNA Polymerase, HC (200 U/µL) (ThermoFisher), 10X RNAPol reaction buffer (1X concentrations: 40 mM Tris-HCl, 6 mM $MgCl_2$, 10mM DTT, 2 mM Spermidine) (NEB), BSA (Molecular Biology Grade, NEB), NTP Set (NEB), $MgCl_2$ (Invitrogen), Tween 20 (Sigma), NEB buffer 3.1 (NEB) were used as received.

DNA oligonucleotides were purchased from Integrated DNA Technologies or Biomers.net with HPLC purification. Stock solutions (100 µM and 10 µM) were made using nuclease-free TE buffer (10 mM Tris, 0.1 mM EDTA, pH 8.0, Integrated DNA Technologies) and stored at -20 °C.

Labelling of BSA with fluorescent dyes

BSA was labelled with fluorescein isothiocyanate (FITC) as follows: 200 mg of BSA was dissolved in 10 mL of 50 mM sodium carbonate buffer (pH 9). 2.36 mg of FITC was dissolved in 590 µL of DMSO and added to the stirred BSA solution. The solution was stirred for 5 h, purified by dialyzing (Medicell dialysis tubing, MWCO 12-14 kDa) overnight against MilliQ water and freeze-dried. BSA was labelled with DyLight 405 as follows: 30 mg of BSA was dissolved in 6 mL of 50 mM sodium carbonate buffer (pH 9). 1 mg of DyLight 405 NHS ester was dissolved in 100 µL of DMF and added to the stirred BSA solution. The solution was stirred for 2 h, purified by dialyzing (Medicell dialysis tubing, MWCO 12-14 kDa) overnight against MilliQ water and freeze-dried.

Synthesis of BSA-NH_2/PNIPAAm nanoconjugates

Cationized BSA (BSA-NH_2) was synthesized according to a previously reported method.[1] Typically, a solution of diaminohexane (1.5g, 12,9 mmol in 10 ml of MilliQ water) was adjusted to pH 6.5 using 5 M HCl and added dropwise to a stirred solution of BSA (200 mg, 3 µmol in 10 ml of MilliQ water). The coupling reaction was initiated by adding 100 mg of 1-(3-dimethylaminopropyl)-3-ethylcarbodiimide HCl (EDC) immediately and then another 50 mg after 5 h. If needed, the pH value was readjusted to 6.5 and the solution was stirred for a further 6 h and then centrifuged to remove any precipitate. The supernatant was dialysed (Medicell dialysis tubing, molecular weight cutoff (MWCO) of 12-14 kDa) overnight against MilliQ water and freeze-dried. End-capped mercaptothiazoline-activated PNIPAAm (Mn = 9,800 g mol-1, 4 mg in 5 ml of MilliQ water) was synthesized according to the previously reported method[1] and added to a stirred solution of BSA-NH_2 (10 mg in 5 ml of PBS buffer at pH 8.0). The solution was stirred for 10 h and then purified using a centrifugal filter (Millipore, Amicon Ultra, MWCO of 50 kDa) and freeze-dried. FITC- and DyLight405-labelled BSA-NH_2/PNIPAAm conjugates were prepared using the same method, except that labelled BSA was used as the starting material.

Preparation of streptavidin-containing proteinosomes

In a typical experiment, BSA-NH_2/PNIPAAm conjugates (final concentration of 8 mg ml^{-1}), streptavidin (final concentrations of 4 or 10 μM) and 0.75 mg of PEG-bis(N-succinimidyl succinate) (Mw = 2,000, approximately 38 PEG units on average) were mixed in 15 μL of 50 mM sodium carbonate buffer (pH 9). A 300 μL volume of 2-ethyl-1-hexanol was immediately added and the mixture was shaken by hand for 20 s to produce a Pickering emulsion. After 2 h of sedimentation, the upper clear oil layer was discarded, 800 μL of 70% ethanol was added and the emulsion was gently shaken. The dispersion was then sequentially dialysed against 70% and 50% ethanol for 2 h each and finally against Milli-Q water for 24 h, resulting in polydisperse microcapsules (size range of 10–60 μm) containing a distribution of encapsulated streptavidin. The proteinosomes were then stored at 4 °C for later use. To produce fluorescently labelled proteinosomes, a 1:7 mixture of labelled (FITC or DyLight 405)/unlabelled BSA-NH_2/PNIPAAm conjugates was used.

DNA and RNA sequence design

DNA sequences were designed by hand with the help of a MATLAB script that generates random sequences with a desired fraction of each nucleotide. The sequences were screened with NUPACK[2] to detect any possible unintended interactions. Nicked T7 promotor regions of the transcriptional switches were based on previous publications.[3] The template strand of the genelet (denoted as **T**) were biotin functionalized to facilitate their binding to proteinosome-localized streptavidin. Transcriptional switches were activated using input strand that hybridize to the single-stranded region of the non-template strand (denoted as **NT**) and thereby complete the promoter region. Monitoring the activation of the transcriptional switches was achieved by either labeling the non-template strand with a fluorophore and the input strand with a corresponding quencher (Figure 1) or by only labeling the input strands with a fluorophore (Figure 3). All biotinylated gate complexes (denoted as **FQ**) in protocells were either labeled with a fluorophore and a corresponding quencher (Figure 1) to observe the activation or only labeled with a fluorophore to detect the strand cleavage (Figure 2). In addition, a nicked DNA target for (d)Cas9 with a removable quencher strand was designed based on earlier work[4] (Figure 3). Sequences of the Malachite Green (MG) genelet and the corresponding RNA aptamer are given in Supplementary Table S1. Sequences of the genelet, RNA signal and the fluorescent probe used in the 2-population signaling cascade are given in Supplementary Table S2. The sequences of double stranded DNA targets for (d)Cas9 and Cas12a with fluorophore and biotin modifications and also the corresponding guide RNA sequences are given in Supplementary Table S3. The sequences of the nicked dCas9 target, the corresponding genelet and the transcribed sgRNA are given in Supplementary Table S4.

sgRNA synthesis

sgRNA strands were synthesized with EnGenTM sgRNA Synthesis Kit, S. pyogenes (NEB), using manufacturer's suggested protocol. The sequences of the target-specific DNA strand and the resulting sgRNA strand are given in Supplementary Table S3-4. Purification was performed with MonarchTM RNA Cleanup Kit (50 µg) (NEB) using manufacturer's suggested protocol. Yield was quantified using NanoDrop One (Thermo Scientific) spectrophotometer.

RNA synthesis

RNA strand were synthesized with T7 RNA Polymerase (ThermoFisher), using manufacturer's suggested protocol. The sequences of the DNA template ($\mathbf{T_1}$, $\mathbf{NT_1}$ and $\mathbf{input_1}$) and the resulting RNA strand ($\mathbf{RNA_1}$) are given in Supplementary Table S2. Purification was performed with MonarchTM RNA Cleanup Kit (50 µg) (NEB) using manufacturer's suggested protocol. Yield was quantified using NanoDrop One (Thermo Scientific) spectrophotometer.

Labeling of T7 RNAP with Cy5

The Tris component in T7 RNAP was replaced with PBS by dialysis. T7 RNAP (4 µM) and Cy5 NHS (8 µM) were mixed in PBS to a final volume of 120 µL. The solution was incubated at room temperature for 45 minutes. Unreacted Cy5 was removed using Amicon Ultra 50kDa 0.5 mL centrifugal filter.

Labeling of dCas9 with Alexa546

Alex546-snap tag substrate was dissolved in fresh DMSO to a final concentration of 250 µM. EnGenTM Spy dCas9 (SNAP-tagTM) (4 µM) and Alexa546-SNAP-tag substrate (8 µM) were mixed in PBS with 1 µM DTT to a final volume of 30 µL. The

solution was incubated in the dark at 37 °C for 30 minutes. Unreacted substrate was removed using Amicon Ultra 50kDa 0.5 mL centrifugal filter.

Localization of genelets, FQ-probes, and DNA targets in streptavidin-containing proteinosomes

The buffer solution for all DNA localization experiments, unless otherwise specified, was 10 mM Tris (pH 8.0, Invitrogen) with 12 mM Mg^{2+} (Invitrogen) and 0.1% vol/vol Tween 20 (Sigma). In a typical localization experiment, 10 µL of a dispersion of streptavidin-containing proteinosomes, 5 µL of 4x buffer and 2 µL of biotinylated DNA duplex (from a 10 µM stock solution) were gently mixed with a pipette in a 1.5 ml Eppendorf tube and incubated at room temperature for 1 h, followed by overnight incubation at 4 °C. The excess unbound DNA strands were removed as follows: 10 µL of the supernatant was carefully removed from the top and discarded, 400 µL of buffer was added and the proteinosomes were resuspended by mixing with a pipette. The proteinosomes were allowed to sediment for 1–2 h or alternatively spun down using a microcentrifuge (4k RCF, for 5 min), then 400 µL of supernatant was removed from the top and discarded. This process was repeated, and the resulting suspension of streptavidin/DNA gate complex-containing proteinosomes was stored at 4 °C.

Cy5-labeled T7 RNAP localization experiments

3 µL of proteinosomes with DNA genelet (Supplementary table S1) or 3 µL empty proteinosomes were suspended in NEB buffer 3.1 and then T7 RNAP-Cy5 (500 nM) was added to a final volume of 10 µL. The suspension was incubated in the dark at 37 °C for 15 minutes and then imaged on glass slide using a confocal laser scanning microscope (CLSM, Leica SP8).

Alexa546-labeled dCas9 localization experiments

3 µL of proteinosomes with DNA target190 (Supplementary table S3) or 3 µL empty proteinosomes were suspended in NEB buffer 3.1 and then dCas9-Alexa546 (500 nM) and sgRNA (1 µM) were added to a final volume of 10 µL. The suspension was incubated in the dark at 37 °C for 15 minutes and then imaged on glass slide using a confocal laser scanning microscope (CLSM, Leica SP8).

Design and fabrication of the microfluidic setup

We used a two-layer microfluidic chip to facilitate the physical trapping and in situ imaging of populations of streptavidin/DNA-loaded proteinosomes. The design of the chip (Figure S16) is based on a previously shown device,[5] but was reduced in size to better facilitate its use in a temperature controlled setup. The chip consisted of a 1.5 mm X 2 mm or 1.5 mm X 1.5 mm localization chamber with PDMS pillars, a filtering chamber, inlet channels with pneumatically actuated Quake style push-up valves and an outlet channel. Master molds for the two layers were fabricated on separate silicon wafers (Silicon Materials) using standard photolithography techniques.[6] The molds for bottom and top layers were made by spin-coating SU8-3050 to a height of 50 µm, and spin-coating AZ 40xt to a height of 40 µm, respectively. After development the AZ 40xt mold was reflowed, resulting in rounded channels with a height of ~60 µm at the center. The microfluidic chips were assembled from PDMS using standard multilayer soft lithography techniques[6] and plasma bonded to rectangular #1.5 glass coverslips.

Proteinosomes experiments in microfluidic devices

The microfluidic chip was inserted into a custom-made PID-controlled temperature control device (Figure S16). The temperature regulator with the microfluidic chip was then mounted on the stage of a confocal laser scanning microscope (CLSM, Leica SP8). Control channels were filled with MilliQ water and actuated using a pneumatic valve array (FESTO), which was in turn actuated using a programmable logic controller (PLC, WAGO Kontakttechnik GmbH). The PLC was connected to the Ethernet port of a PC and controlled using a custom Matlab GUI. The pressure in the control channels was 2 bar. The pressure to the inlet channels was controlled using adjustable pressure regulators (Flow-EZ, Fluigent). In a typical experiment, buffer solution was connected to inlet port 1. First, air bubbles were pushed out of the flow channels by pressurizing the buffer channel at 1 bar and closing all other inlet and outlet valves, followed by thoroughly washing all the flow channels using the buffer solution. Next streptavidin/DNA-containing proteinosomes were loaded into the trap array from inlet port 2 at a pressure of 10 mbar. The inlet port 2 was then closed and the proteinosomes were gently washed (the exact pressure needed to achieve enough flow for effective washing, but without forcing the protocells out of the traps depended on the fluidic resistance of the microfluidic setup and was experimentally determined beforehand; 5 to 15 mbar

was normally used) with buffer solution for 1 to 2 min to remove any unbound DNA. For multi-population experiments, in which it was critical that the unbound DNA was reduced to the minimum to avoid leakage reactions, the populations were loaded sequentially with intermittent washing periods for 1 to 2 min with a pressure of 5 to 15 mbar. To ensure that protocells of different populations were well mixed, additional mixing steps were performed when necessary. Mixing was achieved by first flowing the prococell suspension in reverse direction into one of the input channels and then back into the trapping chamber, this process randomizes the spatial distribution of protocells from different populations. The buffer solution for washing/filling the device and loading/washing the proteinosomes consists of 10 mM Tris (pH 8.0), 12 mM Mg^{2+} and 0.1% v/v Tween 20. The temperature of the device was then set to 37 °C. The confocal microscope was focused on the trapping chamber and time-lapse imaging was started. The initial steady-state signals were recorded as the baseline values.

The reaction buffer for all experiments in a microfluidic device, unless otherwise specified, was 1X NEB RNAPol reaction buffer supplemented with 15 mM $MgCl_2$, 3 mM each NTP and 0.1 mg/mL BSA. Reagents (input DNA, T7 RNAP, Cas12a, Cas9, dCas9) were diluted (and mixed) to the desired concentration in the reaction buffer. The reactions were started by flowing the reagent solution into the trapping chamber using 10 mbar pressure for 20 s. The inlet and outlet valves were then closed and kept closed throughout the experiment.

Data acquisition and analysis

Fluorescence data were acquired using a confocal laser scanning microscope (CLSM, Leica SP8) equipped with solid-state lasers (405 nm for DyLight405, 488 nm for FITC, 552 nm for Cy3 and Alexa546, 638 nm for Cy5) and a hybrid detector. The time-lapse measurements were performed with a ×10/0.40 numerical aperture (NA) (1.55 × 1.55 mm2 field of view, 7 µm slice thickness) objective at a resolution of 512 × 512 pixels. The photon counting mode of the hybrid detector was used.

The RFU-to-concentration conversion factor was determined by measuring the average RFU (value across a horizontal line through the device) of specific DNA gate complex (0.25 µM, 0.5 µM, 1 µM and 2 µM) that was flown into the device. The conversion factor was then determined by plotting the RFU vs the concentration of DNA gate complex (Figure S17).

Zeta potential measurement

All measurements (Figure S1) were performed using a Malvern zetasizer nano-ZS instrument with a temperature controller. The samples (0.1 mg/ml) were measured in PBS buffer (pH 7.4, 0.1 mM) at room temperature.

Gel electrophoresis

Native Polyacrylamide gel electrophoresis (native-PAGE) was used to confirm the production of RNA from genelet circuit encoding aptamer sequence (Figure S2). Gels was prepared at 12% monomer concentration of acrylamide in gel buffer (44.5 mM Tris, 44.5 mM Boric acid, 11.5mM MgCl2, pH 8.0). The gel was run in gel buffer for 1.5 h at 150 V at room temperature and post-stained with SYBR Gold. GeneRuler DNA ladder was included as a reference. Gels were imaged using an ImageQuant 400 Digital Imager (GE Healthcare).

Fluorescence measurements on a plate reader

Batch testing of the fluorescent malachite green aptamer transcription (Figure S2), genelet-based activation of $\mathbf{F_1Q_1}$ probe (Figure S5), (d)Cas9 probe (Figure S11) and genelet-based activation of dCas9 (Figure S14) were performed at 37 °C on a Biotek Synergy H1m plate reader using 384 well low-volume plates (Nunc) with a reaction volume of 12 µL. Excitation and emission wavelengths were 640 nm and 681 nm respectively. Fluorescence measurements were performed every 30 s.

Supplementary Figures

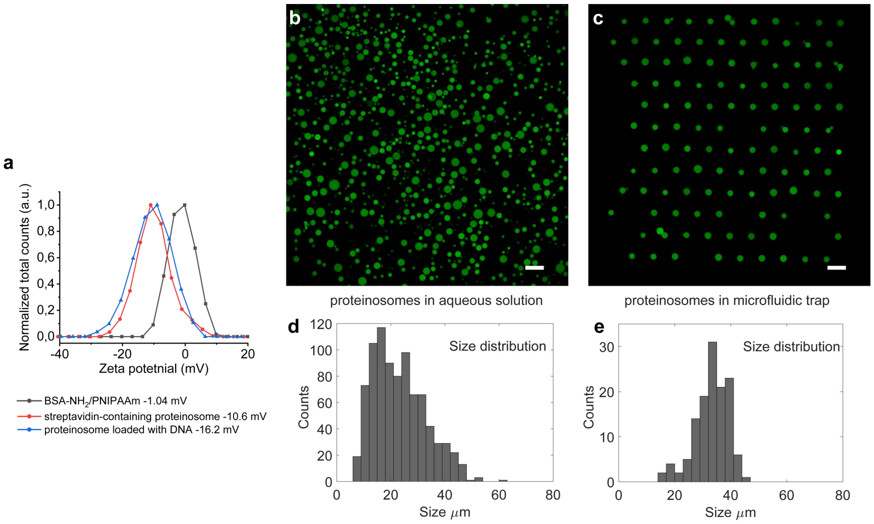

Supplementary Figure S1. Zeta potentials and size distributions of the protocells. a) The BSA-NH_2/PNIPAAm nanoconjugates have a zeta potential of -1.04 mV (black). The streptavidin-containing proteinosome has a lower zeta potential (-10.6 mV, red) because the BSA-NH_2 molecules in the proteinosome membrane were cross-linked resulting in reduction in the number of surface amine groups. The charge implies that RNA/DNA do not electrostatically bind to the membrane and diffuse passively into the lumen. The zeta potential of proteinosome further decreased after localization of negatively charge DNA (-16.2 mV, blue). The samples (0.1 mg/ml) were measured in PBS buffer at room temperature. Confocal fluorescence micrographs of FITC labeled proteinosomes in an aqueous solution on a glass slide (b) and microfluidic trapping device (c). Scale bars 100 μm. The size distributions are shown in (d) and (e), respectively. The size distribution become narrower after loading into the microfluidic device because the very small protocells can not be trapped and the protocells with a very large size are trapped in the filter chamber.

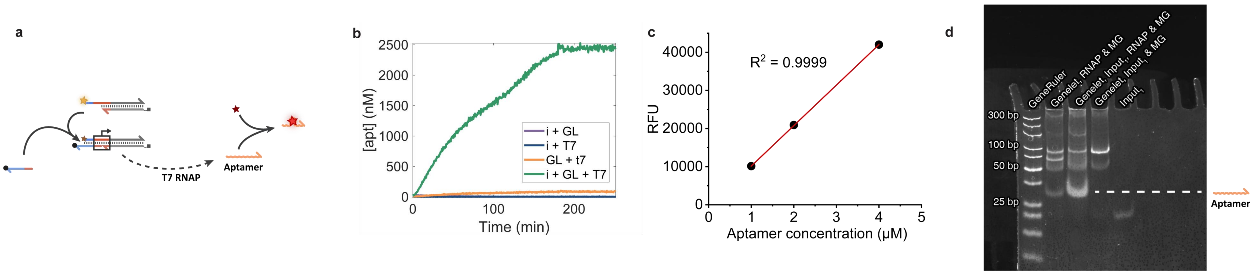

Supplementary Figure S2. Malachite Green (MG) aptamer transcription in batch conditions. a) Reaction diagram of malachite green aptamer transcription using a genelet module in batch conditions. b) The fluorescence of MG was measured and converted to concentration of the transcribed aptamer. The RFU-to-concentration conversion factor was determined by fluorescence measurement of 20 μM MG with a range of concentrations (1, 2 and 4 μM) of aptamer at 681 nm (excitation wavelength 640 nm). The data was fitted through a straight line and coefficient of determination (R^2) was calculated (c). A significant increase in MG fluorescence was observed only when the genelet (GL), input (i) and T7 RNA polymerase (T7) were all present (green trace). Experiments were performed in reaction buffer with 200 nM input, 100 nM genelet, 2 U/μL T7 RNAP and 20 μM MG at 37°C (supplementary methods, plate reader). d) After fluorescence measurement the samples were analyzed by native-PAGE gel (12% polyacrylamide, stained for DNA with SYBR Gold). The image observed low level leaky transcription of T7 RNAP and genelet without input (second lane, aptamer was indicated by dash line) and confirmed the significant production of RNA from the complete genelet circuit in the presence of T7 RNAP (third lane).

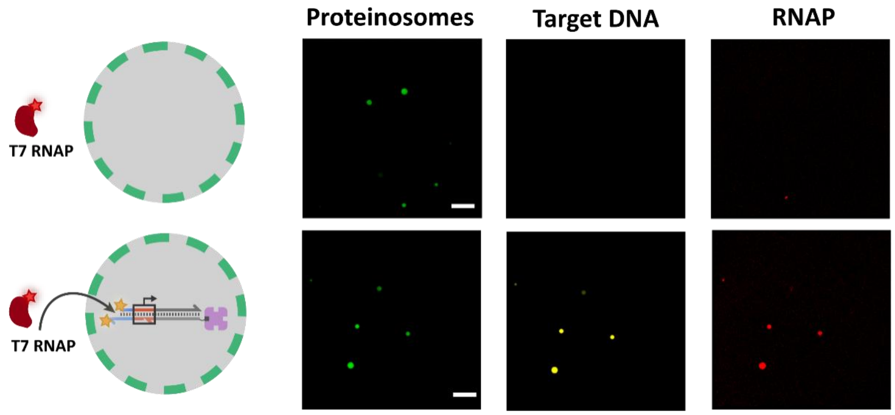

Supplementary Figure S3. Fluorescent T7 RNAP binding to proteinosome-localized genelet target. Reaction diagram of T7 RNAP localization strategy. A Cy3-labeled genelet dsDNA target (yellow) with a biotin modification was encapsulated in streptavidin-containing proteinosomes (FITC, green). Confocal fluorescence micrographs of the empty and target-containing proteinosomes populations after incubating with Cy5-T7 RNAP (500 nM) in reaction buffer for 0.5 h at 37°C showed significant localization (red) only in target-containing proteinosomes. Scale bars 200 µm.

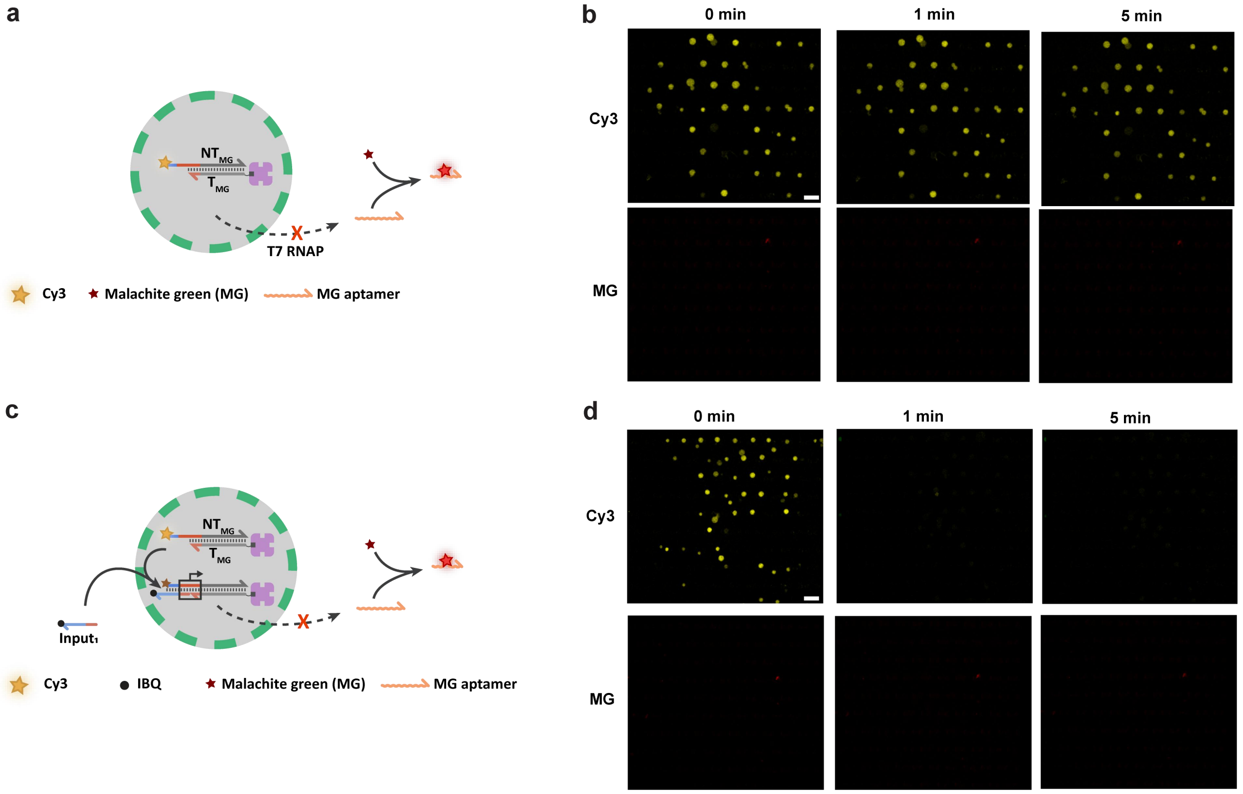

Supplementary Figure S4. MG system in microfluidic trapping device without input or without T7 RNAP. a) Reaction diagram of the MG aptamer-transcribing protocells of Figure 1c in the absence of the input strand. Experiments were performed in reaction buffer with T7 RNAP (5 U/µL) and MG (20 µM). b) Confocal fluorescence micrographs of MG aptamer-transcribing protocells in the absence of input showed no change in Cy3 fluorescence (yellow) and MG fluorescence (red) indicating no genelet activation and RNA aptamer production. c) Reaction diagram of the MG aptamer-transcribing protocells of Figure 1c in the absence of T7 RNAP. Experiments were performed in reaction buffer with **Input$_1$** (500 nM), MG (20 µM). d) Confocal fluorescence micrographs of MG aptamer-transcribing protocells in the absence of T7 RNAP showing a decrease in Cy3 fluorescence (yellow) associated with genelet activation, but no increase in MG fluorescence (red) indicated no RNA aptamer production. Scale bars 100 µm. All experiments were performed at 37°C.

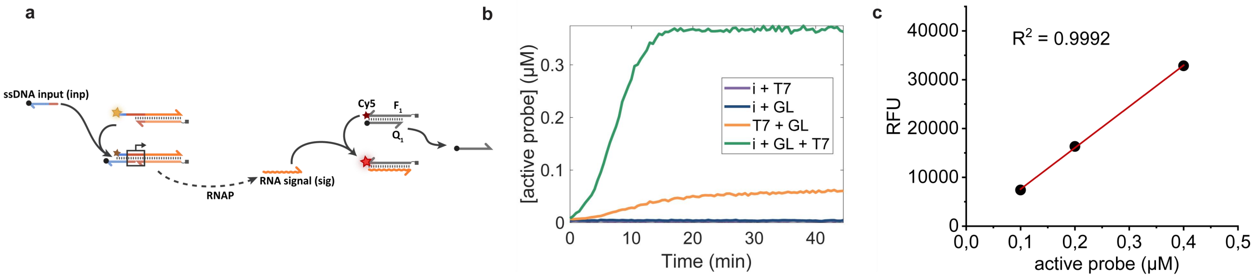

Supplementary Figure S5. RNA-based signaling cascade in batch conditions. a) Reaction diagram of the RNA mediated signaling cascade of Figure 1e in batch conditions. b) Concentration of the activated $\mathbf{F_1Q_1}$ probe. A significant amount of probe activation was observed only in the presence of the genelet (GL), input (i) and T7 RNA polymerase (T7) together (green trace). Small amount of activation due to leakage of the genelet module was also observed in the absence of the input strand (orange trace). Experiments were performed in reaction buffer with 200 nM input, 100 nM genelet, 2 U/µL T7 RNAP and 500 nM $\mathbf{F_1Q_1}$ probe at 37°C (supplementary methods, plate reader). c) The RFU-to-concentration conversion factor was determined by fluorescence measurement over a range of concentrations (0.1, 0.2 and 0.4 µM) of active probes at 681 nm (excitation wavelength 640 nm). The data was fitted through a straight line and coefficient of determination (R^2) was calculated.

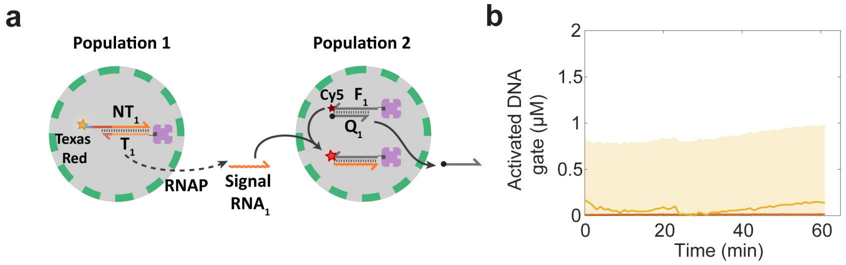

Supplementary Figure S6. Control experiment of the two-population signaling cascade in the absence of input. a) Reaction diagram of the RNA mediated signaling cascade of Figure 1e in the absence of the input strand. b) Means and standard deviations of the activated concentrations of two proteinosome populations without input in a microfluidic trapping device. Yellow line corresponds to Texas Red in population **1** and Red line corresponds to Cy5 in population **2**. The number of analyzed proteinosomes was 48. Fractions of the two populations were q1 = 0.68 and q2 = 0.32, respectively. Experiment was performed in reaction buffer with 5 U/ µL of T7 RNAP (37°C).

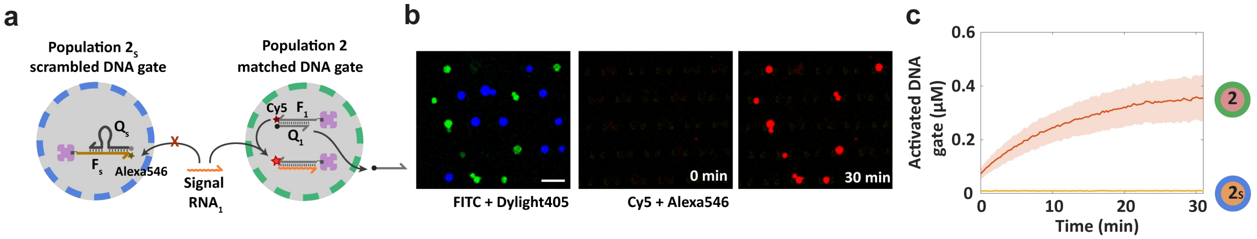

Supplementary Figure S7. RNA signal only activates protocell-encapsulated DNA gates with correct sequences. a) Reaction diagram of adding RNA signal to protocells containing $\mathbf{F_1Q_1}$ (correct sequence) or $\mathbf{F_sQ_s}$ (scrambled sequence). b) Confocal fluorescence micrographs of population **2** (FITC-labeled proteinosome membrane, green) and $\mathbf{2_s}$ protocells (Dylight405-labeled proteinosome membrane, blue) showing time-dependent increase of Cy5 fluorescence (red) in population **2** and no increase of Alexa546 fluorescence (yellow) in population $\mathbf{2_s}$. Scale bar 100 µm. c) Means and standard deviations of the activated concentrations of these two proteinosome populations upon addition of RNA signal (1 µM) in a microfluidic trapping device. Red line corresponds to Cy5 in population **2** and yellow line corresponds to Alexa546 in population $\mathbf{2_s}$. Only population **2** with correct DNA sequence was activated showing target specificity. The numbers of analyzed population **2** and $\mathbf{2_s}$ was 36 and 48, respectively. Experiment was performed at room temperature.

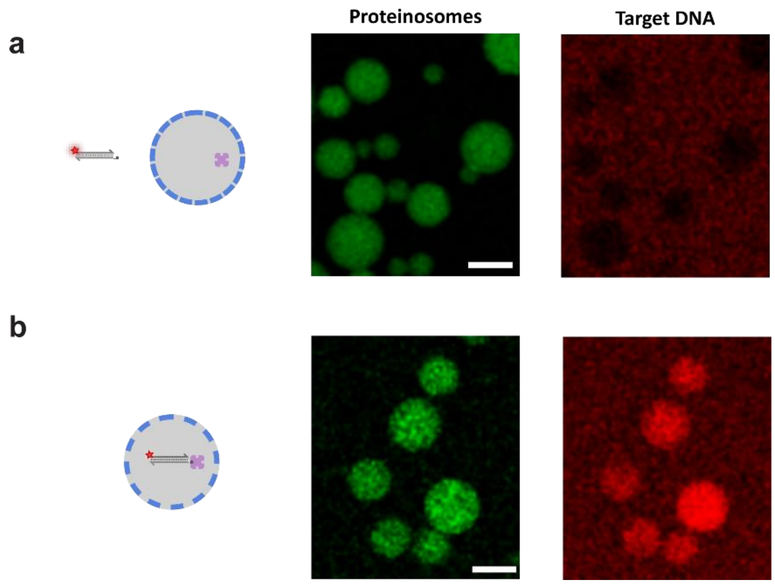

Supplementary Figure S8. **Proteinosomes prepared with different nanoconjugate concentrations displayed distinct localization behaviors of 190mer dsDNA.** Streptavidin-containing proteinosomes (FITC, green) prepared with high (a, 16 mg/ml) and low (b, 8 mg/ml) nanoconjugate BSA-NH_2/PNIPAAm concentrations were mixed with 190mer biotinylated dsDNA (Cy5, red) with a final concentration of 200 nM in reaction buffer after 5 hours at 4 °C. Localization of 190mer dsDNA was only observed in **b**. Scale bars 50 μm.

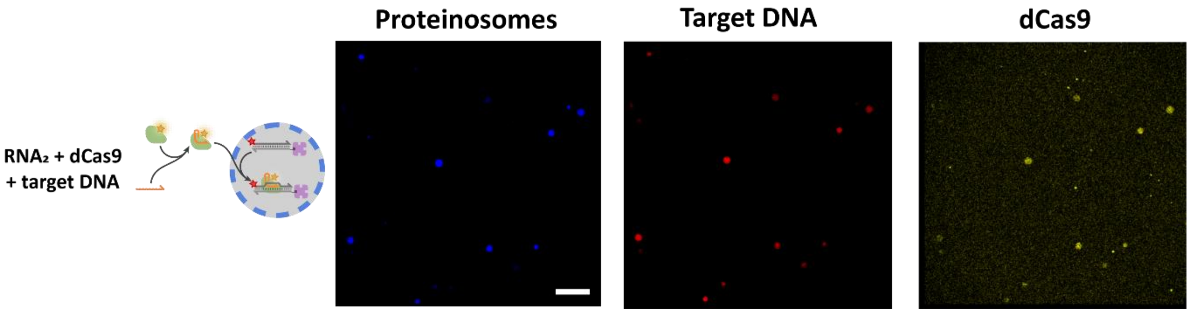

Supplementary Figure S9. Fluorescent dCas9 binding to proteinosome-encapsulated dsDNA target. The blue channel corresponds to the dylight405 fluorophore on the BSA-polymer building blocks and showed all the proteinosomes present. Red (Cy5) channel showed the localized dsDNA target. The yellow channel corresponds to the Alexa546-labeled dCas9 showing different dCas9 localization levels due to differences in proteinsome permeabilities. Experiment was performed in reaction buffer with Alexa546-dCas9 (500 nM) and sgRNA strand RNA_2 (1 μM) for 15 mins at 37°C. Scale bar 100 μm.

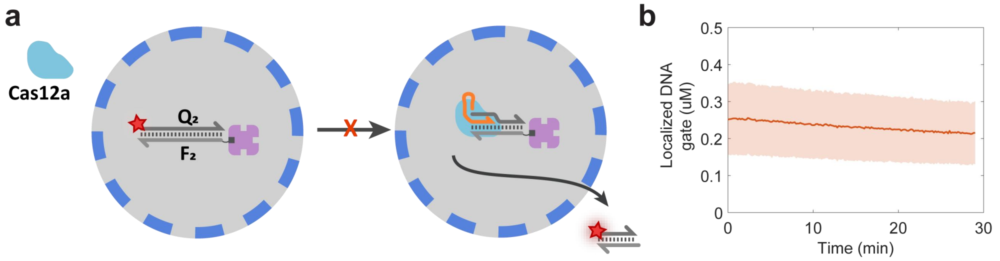

Supplementary Figure S10. Control experiment of the Cas12a-mediated cleavage of DNA target in the absence of sgRNA. a) Reaction diagram of the Cas12a-based cleavage of proteinosome-encapsulated DNA target of Figure 2c in the absence of the sgRNA **RNA_3**. b) Mean and standard deviation of the Cy5 fluorescence signals of a proteinosome population where only Cas12a was added. The number of analyzed proteinosomes was 42. Experiment was performed in reaction buffer with Cas12a (1 μM) at 37°C.

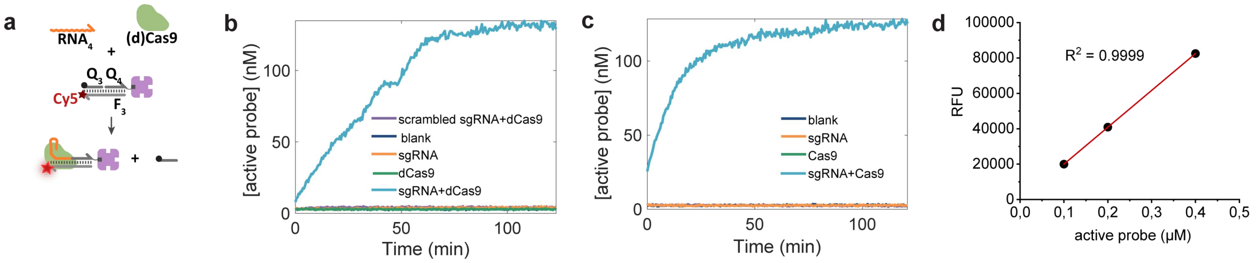

Supplementary Figure S11. Activation of fluorescent (d)Cas9 probe in batch conditions. a) Molecular reaction diagram of the probe. Concentrations of the activated fluorescent probe using dCas9 (b) or Cas9 (c) in different experimental conditions. In all experiments the concentrations of probe was 500 nM. Activation was only observed when (d)Cas9 (500 nM) and the corresponding sgRNA strand **RNA_4** (500 nM) were both present. A scrambled sgRNA (500 nM) together with dCas9 (500 nM) were used as control experiments. No (d)Cas9 and sgRNA were added in the blank. All experiments were performed in reaction buffer at 37°C (supplementary methods, plate reader). c) The RFU-to-concentration conversion factor was determined by fluorescence measurement over a range of concentrations (0.1, 0.2 and 0.4 μM) of active probes at 681 nm (excitation wavelength 640 nm). The data was fitted through a straight line and coefficient of determination (R^2) was calculated.

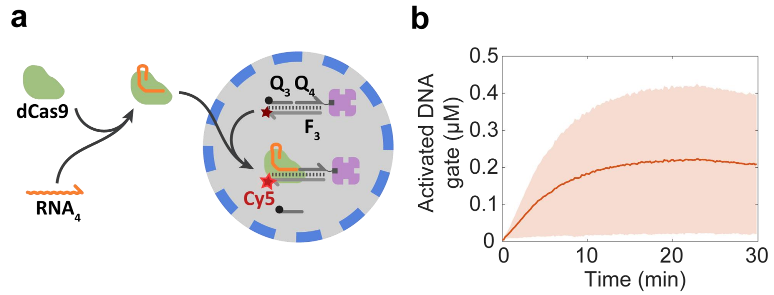

Supplementary Figure S12. dCas9-localizing fluorescence assay implemented in proteinosomes in microfluidic device. a) Molecular reaction diagram of the dCas9-localizing fluorescence probe in proteinosomes. b) Fluorescence data showing the time-dependent activation of the Cy5 labeled probe in a population of proteinosomes in a microfluidic trapping device. The number of analyzed proteinosomes was 69. Experiment was performed in reaction buffer with 1 μM sgRNA and 500 nM dCas9 (37°C.).

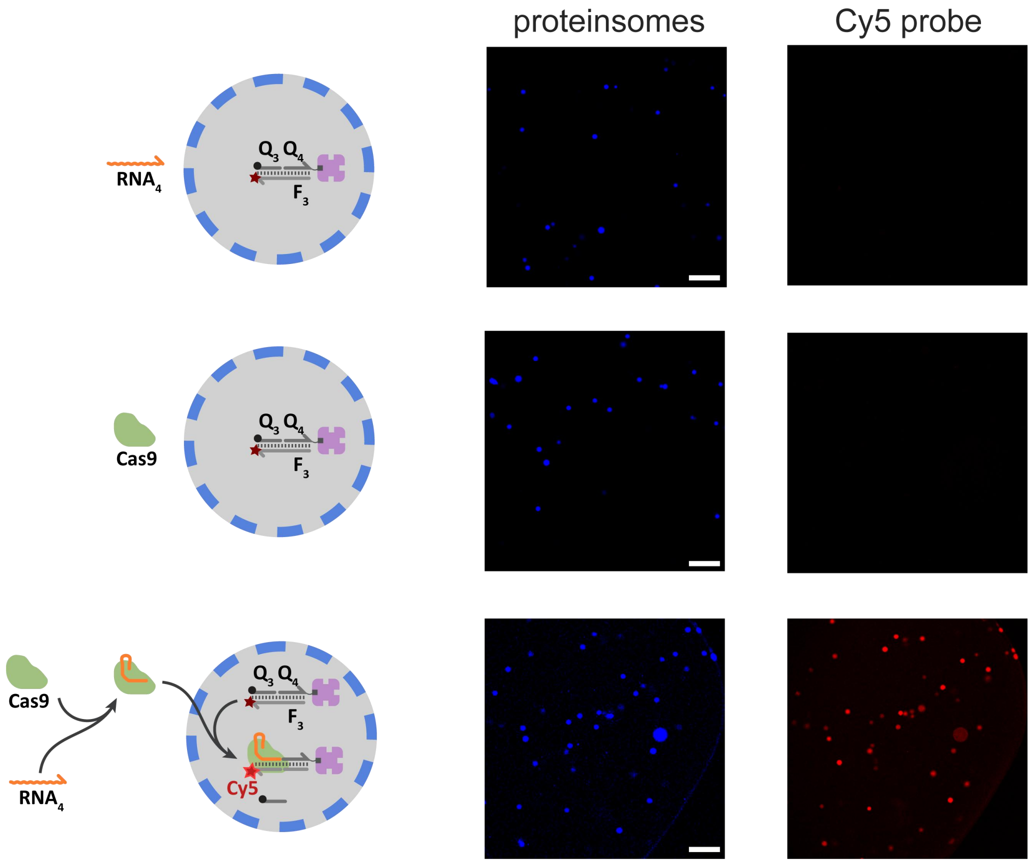

Supplementary Figure S13. Cas9-localizing fluorescence assay implemented in proteinosomes on glass slide. Molecular reaction diagram of the Cas9-localizing fluorescence probe in proteinosomes. Confocal fluorescence micrographs showed the activation of the Cy5 labeled probe in a population of proteinosomes was only observed when both RNA and Cas9 were present. Experiment was performed in reaction buffer with 1 μM sgRNA and 500 nM Cas9 (37°C.). Scale bar 200 μm.

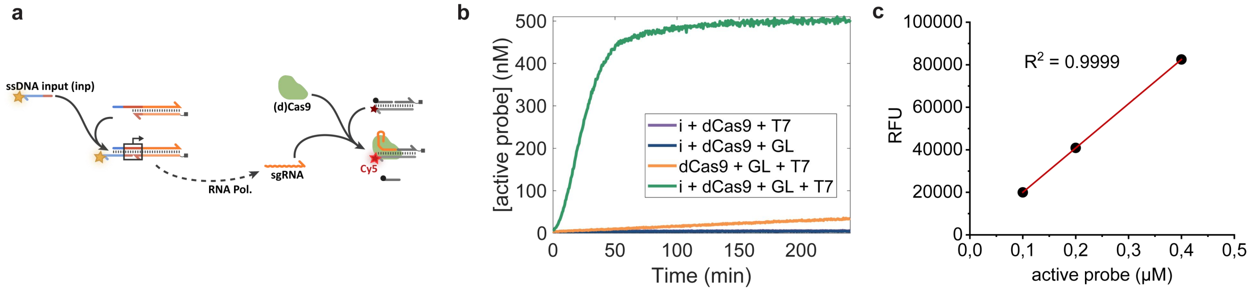

Supplementary Figure S14. Genelet-triggered activation of dCas9 in batch conditions. a) Reaction diagram of the genelet-controlled dCas9 activation in batch. b) Concentrations of the activated $\mathbf{F_3F_4Q_3}$ probe. A significant amount of probe activation was observed only in the presence of the genelet (GL), input (i), T7 RNA polymerase (T7) and dCas9 together (green trace). Small amount of activation due to leakage of the genelet module is also observed in the absence of the input strand (orange trace). Experiments were performed in reaction buffer with 200 nM $\mathbf{input_2}$, 100 nM genelet, 2 U/µL T7 RNAP, 500 nM $\mathbf{F_3F_4Q_3}$ probe and 500 nM dCas9 at 37°C (supplementary methods, plate reader). c) The RFU-to-concentration conversion factor was determined by fluorescence measurement over a range of concentrations (0.1, 0.2 and 0.4 µM) of active probes at 681 nm (excitation wavelength 640 nm). The data was fitted through a straight line and coefficient of determination (R^2) was calculated.

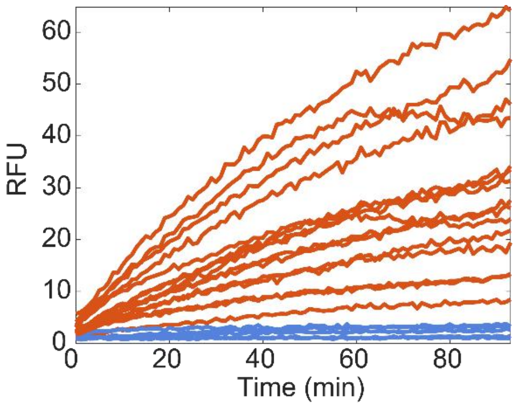

Supplementary Figure S15. Individual fluorescence traces in protocells in Figure 3c. The traces (blue lines) that did not show any activation were excluded from analysis.

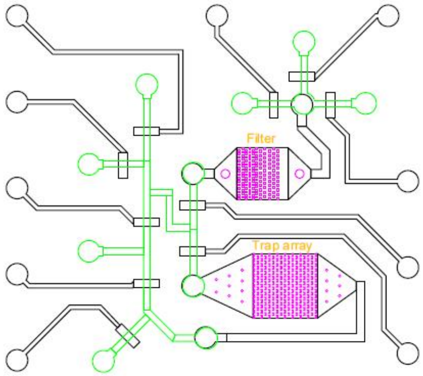

Supplementary Figure S16. Microfluidic design. CAD drawing of the microfluidic device adapted from a previously developed chips.[5] Trap array and filter are in purple. Channels of the bottom layer (bonded to the glass slide) are in black. The rounded top layer channels are in green. The flow channels cross from the top layer to the bottom layer as only the rounded channels of the top layer can be closed using the pneumatic valves, while the trapping array requires the rectangular channels of the bottom layer.

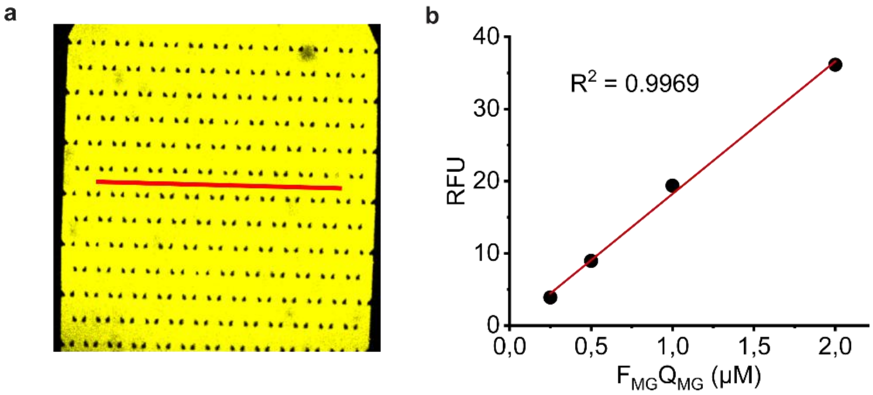

Supplementary Figure S17. Verification of the linearity of the concentration estimation method. a) A confocal micrograph of the microfluidic trapping device filled with inactivated genelet gate complex $F_{MG}Q_{MG}$ (2 µM). The corresponding RFU was obtained by measuring the average RFU value across a straight horizontal line (red) through the device. b) The linearity of the conversion method was verified by performing the measurements shown in a range of concentrations (250 nM, 0.5 µM, 1 µM and 2 µM) of $F_{MG}Q_{MG}$, fitting a straight line through the data and calculating the coefficient of determination (R^2).

Supplementary Tables

Supplementary Table S1: DNA/RNA sequences of malachite green aptamer transcription. Promoter region is marked in green.

		Length bases	5' modification	3' modification
Figure 1c, Figure S2, S3, S4				
T_{MG}	GATCCATTCGTTACCTGGCTCTCGCCAGTCGGG ATCCCTATAGTGAGTCG	50	Biotin	
NT_{MG}	CATTAGTGTCGTTCGTTCACAGTAATACGACTCA CTATAGGGATCCCGACTGGCGAGAGCCAGGTAA CGAATGGATC	77	Cy3	
$Input_1$	TATTACTGTGAACGAACGACACTAATGAACTACT AC	36		Lowa Black quencher
MG aptamer	GGGAUCCCGACUGGCGAGAGCCAGGUAACGAA UGGAUC	38		

Supplementary Table S2: DNA/RNA sequences of RNA signaling cascade. Promoter region is marked in green.

		Length Bases	5' modification	3' modification
Figure 1e, Figure S5, S6,S7				
T_1	TTTCTGACTTTGTCAGTATTATCATTCCATCTTAC CCTTGCTTCAATCCGTTTTACTCTCCCTATAGTGA GTCG	74	Biotin	
NT_1	CATTAGTGTCGTTCGTTCACAGTAATACGACTCA CTATAGGGAGAGTAAAACGGATTGAAGCAAGGG TAAGATGGAATGATAATACTGACAAGTCAGAAA	101	Texas Red	
$Input_1$	TATTACTGTGAACGAACGACACTAATGAACTACT AC	36		Lowa Black quencher
Signal (RNA_1)	GGGAGAGUAAAACGGAUUGAAGCAAGGGUAAG AUGGAAUGAUAAUACUGACAAAGUCAGAAA	62		
F_1	TTTTTTTTATCATTCCATCTTACCCTTGCTTCAATC CGTTTTACTCTCCC	50	Biotin	Cy5
Q_1	GGGAGAGTAAAACGGATTGAAGCAAGGGTAAGA TGG	36	Lowa Black quencher	
F_s (scrambled)	GTAGTAGTGCATTAGTCTATCATGGTCGTTCGTT CG	36	Biotin	Alexa546
Q_s (scrambled)	CGAACGAACGACCATGCGTGAAACATAGACTAA TGC	36	Lowa Black quencher	

Supplementary Table S3: DNA sequences targeted by Cas12a/(d)Cas9 and RNA sequences.

		Length Bases	5' modification	3' modification
Figure 2a, Figure S8, S9				
Q_2	TTTTTTTTTTTGCGTCGAAACTGAAACTCCTGCAATG<br>GTGGTGGATAGACTCGTAGATAATGAAATGGCAATG<br>GTGGTGGTAAGACTCAGAATGGTAGATAAGGCAATG<br>GTGGTGGTTAGACTCAGAAGGAAGAAGAAGGCAAT<br>GGTGGTGGGAAGAGCTGAGAGGACCTAGTGAGACA<br>AAAAAAAAAAA	190	Biotin	
F_2	TTTTTTTTTTTTGTCTCACTAGGTCCTCTCAGCTCTTC<br>CCACCACCATTGCCTTCTTCTTCCTTCTGAGTCTAAC<br>CACCACCATTGCCTTATCTACCATTCTGAGTCTTACC<br>ACCACCATTGCCATTTCATTATCTACGAGTCTATCCA<br>CCACCATTGCAGGAGTTTCAGTTTCGACGCAAAAAA<br>AAAAA	190	Cy5	
RNA_2	GUGGUGGUGGGAAGAGCUGAGGUUUUAGAGCUAG<br>AAAUAGCAAGUUAAAAUAAGGCUAGUCCGUUAUCA<br>ACUUGAAAAAGUGGCACCGAGUCGGUGCUUUU	101		
scrambled RNA	GAAGAAGAAGGCAAUGGUGGGUUUUAGAGCUAGA<br>AAUAGCAAGUUAAAAUAAGGCUAGUCCGUUAUCAA<br>CUUGAAAAAGUGGCACCGAGUCGGUGCUUUU	100		
Figure 2c, Figure S10				
Q_2	TTTTTTTTTTTGCGTCGAAACTGAAACTCCTGCAATG<br>GTGGTGGATAGACTCGTAGATAATGAAATGGCAATG<br>GTGGTGGTAAGACTCAGAATGGTAGATAAGGCAATG<br>GTGGTGGTTAGACTCAGAAGGAAGAAGAAGGCAAT<br>GGTGGTGGGAAGAGCTGAGAGGACCTAGTGAGACA<br>AAAAAAAAAAA	190	Biotin	
F_2	TTTTTTTTTTTTGTCTCACTAGGTCCTCTCAGCTCTTC<br>CCACCACCATTGCCTTCTTCTTCCTTCTGAGTCTAAC<br>CACCACCATTGCCTTATCTACCATTCTGAGTCTTACC<br>ACCACCATTGCCATTTCATTATCTACGAGTCTATCCA<br>CCACCATTGCAGGAGTTTCAGTTTCGACGCAAAAAA<br>AAAAA	190	Cy5	
RNA_3	UAAUUUCUACUAAGUGUAGAUUCUCACUAGGUCCU<br>CUCAG	40		

Supplementary Table S4: DNA/RNA sequences of genelet-controlled (d)Cas9 localization and activation. Promoter region is marked in green.

		Length Bases	5' modification	3' modification
Figure 3, Figure S11-S15				
NT_2	TTAGTGTCGTTCGTTCACAGTAATACGACTCACTATA<br>GTGGTGGTGGGAAGAGCTGAGGTTTTAGAGCTAGA<br>AATAGCAAGTTAAAATAAGGCTAGTCCGTTATCAACT<br>TGAAAAAGTGGCACCGAGTCGGTGCTTTT	138	Biotin	
T_2	AAAAGCACCGACTCGGTGCCACTTTTTCAAGTTGAT<br>AACGGACTAGCCTTATTTTAACTTGCTATTTCTAGCT<br>CTAAAACCTCAGCTCTTCCCACCACCACTATAGTGA<br>GTCG	113		
$Input_2$	TATTACTGTGAACGAACGACACTAATGAACTACTAC	36		Cy3
RNA_4	GUGAAGUGUCCAUAAAUGAGGUUUUAGAGCUAGAA<br>AUAGCAAGUUAAAAUAAGGCUAGUCCGUUAUCAAC<br>UUGAAAAAGUGGCACCGAGUCGGUGCUUUU	100		
scrambled RNA	GAAGAAGAAGGCAAUGGUGGGUUUUAGAGCUAGA<br>AAUAGCAAGUUAAAAUAAGGCUAGUCCGUUAUCAA<br>CUUGAAAAAGUGGCACCGAGUCGGUGCUUUU	100		
Q_4	AGGACCTAGTGAGACAAAAAAAAAAAA	27		Biotin
Q_3	CAATGGTGGTGGGAAGAGCTGAG	23	Lowa Black quencher	
F_3	TTTTTTTTTTTTGTCTCACTAGGTCCTCTCAGCTCTTC<br>CCACCACCATTG	50		Cy5

Supplementary References

[1] X. Huang, M. Li, D. C. Green, D. S. Williams, A. J. Patil, S. Mann, *Nat. Commun.* **2013**, *4*, 2239.

[2] J. N. Zadeh, C. D. Steenberg, J. S. Bois, B. R. Wolfe, M. B. Pierce, A. R. Khan, R. M. Dirks, N. A. Pierce, *J. Comput. Chem.* **2011**, *32*, 170–173.

[3] J. Kim, K. S. White, E. Winfree, *Mol. Syst. Biol.* **2006**, *2*, 68.

[4] V. Mekler, L. Minakhin, E. Semenova, K. Kuznedelov, K. Severinov, *Nucleic Acids Res.* **2016**, *44*, 2837–2845.

[5] A. Joesaar, S. Yang, B. Bögels, A. van der Linden, P. Pieters, B. P. Kumar, N. Dalchau, A. Phillips, S. Mann, T. F. A. de Greef, *Nat. Nanotechnol.* **2019**, *14*, 369–378.

[6] M. A. Unger, H.-P. Chou, T. Thorsen, A. Scherer, S. R. Quake, *Science.* **2000**, *288*, 113–116.
